# Wafer-Scale Ag_2_S-Based Memristive Crossbar Arrays with Ultra-Low Switching-Energies Reaching Biological Synapses

**DOI:** 10.1007/s40820-024-01559-2

**Published:** 2024-11-22

**Authors:** Yuan Zhu, Tomas Nyberg, Leif Nyholm, Daniel Primetzhofer, Xun Shi, Zhen Zhang

**Affiliations:** 1 Division of Solid-State Electronics, Department of Electrical Engineering, Uppsala University, 75121 Uppsala, Sweden; 2 Department of Chemistry, Uppsala University, Uppsala, Sweden; 3 Department of Physics and Astronomy, Uppsala University, Uppsala, Sweden; 4 State Key Laboratory of High Performance Ceramics and Superfine Microstructure, Shanghai Institute of Ceramics, Chinese Academy of Sciences, Shanghai, 200050 People’s Republic of China

**Keywords:** Wafer-scale Ag_2_S films, Reactive sputter, Silver nucleation, Ag^+^ migration, Energy-efficient neuromorphic computing

## Abstract

Memristive crossbar arrays (MCAs) offer parallel data storage and processing for energy-efficient neuromorphic computing. However, most wafer-scale MCAs that are compatible with complementary metal-oxide-semiconductor (CMOS) technology still suffer from substantially larger energy consumption than biological synapses, due to the slow kinetics of forming conductive paths inside the memristive units. Here we report wafer-scale Ag_2_S-based MCAs realized using CMOS-compatible processes at temperatures below 160 °C. Ag_2_S electrolytes supply highly mobile Ag^+^ ions, and provide the Ag/Ag_2_S interface with low silver nucleation barrier to form silver filaments at low energy costs. By further enhancing Ag^+^ migration in Ag_2_S electrolytes via microstructure modulation, the integrated memristors exhibit a record low threshold of approximately − 0.1 V, and demonstrate ultra-low switching-energies reaching femtojoule values as observed in biological synapses. The low-temperature process also enables MCA integration on polyimide substrates for applications in flexible electronics. Moreover, the intrinsic nonidealities of the memristive units for deep learning can be compensated by employing an advanced training algorithm. An impressive accuracy of 92.6% in image recognition simulations is demonstrated with the MCAs after the compensation. The demonstrated MCAs provide a promising device option for neuromorphic computing with ultra-high energy-efficiency.

**Supplementary Information:**

The online version contains supplementary material available at 10.1007/s40820-024-01559-2.

## Highlights

- Wafer-scale integration of Ag_2_S-based memristive crossbar arrays was demonstrated using complementary metal–oxide–semiconductor (CMOS) compatible processes below 160 °C.
- A record-low threshold voltage for filament formation and an ultra-low switching-energy reaching that of biological synapses in wafer-scale CMOS-compatible memristive units were achieved.
- The energy-efficient resistance switching was enabled by self-supply of mobile Ag^+^ ions in Ag_2_S electrolytes and low silver-nucleation barrier at Ag/Ag_2_S interface.

**Supplementary Information:**

The online version contains supplementary material available at 10.1007/s40820-024-01559-2.

## Graphical abstract

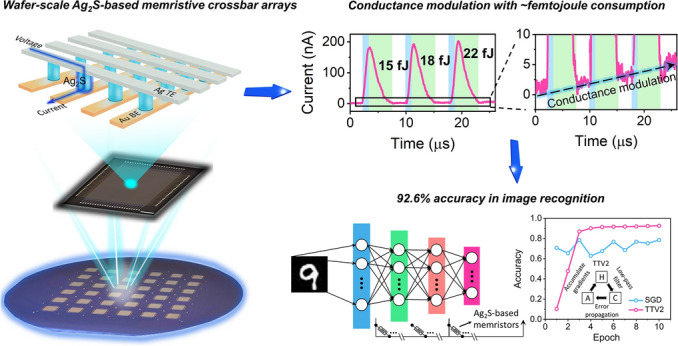

## Introduction

As artificial neural networks grow in scale and complexity, traditional von Neumann computational architectures with separate memory and processing units are becoming increasingly inadequate to train these models. The frequent data shuttling between storage and processing units creates severe time and energy demands during computation [1, 2]. To address this challenge, memristor-based in-memory computing systems are being explored as a promising alternative [3]. Memristors including phase change memories (PCMs) [4], conductive-bridging memories (CBMs) [5], resistive random-access memories (RRAMs) [6, 7], electrochemical random-access memories (ECRAMs) [8–13] and phototransistor memories [14], can function as artificial synapses to realize parallel data storage and processing [15–17]. However, most of these device options still face significant technical challenges, notably non-ideal switching characteristics, incompatibility with complementary metal–oxide–semiconductor (CMOS) technology, and high energy consumptions. Non-ideal switching characteristics, such as asymmetric and nonlinear conductance modulation, switching variability, and reading noise, degrade the precision of synaptic weight update during deep learning neural network (DNN) training. Poor compatibility with CMOS technology hinders the large-scale integration of memristive crossbar arrays (MCAs) required for complex computing tasks. Moreover, the substantially higher energy consumption compared to biological synapses (which consume approximately 1–100 fJ per synaptic event) will significantly undermine the energy efficiency of MCA-based computing hardware, and limit its applications in energy-constrained environments (such as portable electronics). Although the impact of non-ideal memristive behavior could be mitigated by advanced training algorithms [18, 19], achieving an energy efficiency that is comparable to biological synapses remains challenging for CMOS-compatible MCAs. In most memristors, resistance switching (RS) relies on material transformations inside solid electrolytes, such as phase transition in PCMs, filament formation in RRAMs and CBMs, and ionic intercalation in ECRAMs. Slow kinetics of atomic or ionic movements for material transformations is the fundamental cause of high energy consumptions in these devices. While some memristors based on 2D materials demonstrate relatively low switching-energies, their fabrication often involves complex mechanical exfoliation and substrate transfer processes, as well as high temperature treatments (often approaching 1000 °C) to obtain high-quality monolayers [20, 21]. These processes not only often lead to folded and wrinkled film flakes with severe morphological irregularities, but also exceed the thermal budget limitation for the back-end-of-line integration with CMOS circuitry [22, 23]. Developing MCAs with energy-efficient RS characteristics and CMOS-compatible processes is therefore greatly desired.

Monoclinic Ag_2_S exhibits both electron and ion conductivity, as well as metal-like ductility at room temperature [24]. Due to its liquid-like silver sublattices, Ag_2_S provides abundant mobile silver ions that can potentially facilitate energy-efficient silver filament formation inside Ag_2_S memristors. However, previous studies on memristive behavior of Ag_2_S devices were limited to material evaluation or stand-alone devices on bulk Ag_2_S substrates prepared using ceramic processes [25–29]. These Ag_2_S devices are not compatible with CMOS fabrication technologies. Wafer-scale integration of Ag_2_S MCAs has not been reported, either. In this work, we used fully CMOS-compatible processes with a thermal budget below 160 °C to realize wafer-scale integration of Ag_2_S-based MCAs. The integrated memristor consumes only femtojoules per writing operation, demonstrating ultra-low energy consumptions reaching those of biological synapses. We reveal that energy-efficient silver filament formation is enabled by the self-supply of highly mobile Ag^+^ ions in Ag_2_S, together with the low silver nucleation barrier at the Ag/Ag_2_S interface. Moreover, with low thermal budget fabrication, the same MCAs were also realized on polymer substrates, and demonstrated on-chip multiply accumulate calculations capabilities. The flexible array also yielded an impressive image classification accuracy of 92.6%, with the inherent non-ideal switching characteristics being compensated by an advanced training algorithm.

## Experimental Section

### Synthesis of Ag_2_S Films

Ag_2_S films were synthesized utilizing reactive sputtering. Before deposition, the chamber was evacuated by turbo pumping to reach a high vacuum of ~ 10^–7^ Torr. A mixture of Ar (20 sccm) and H_2_S (40 sccm) gases was subsequently introduced into the sputter chamber, with the chamber pressure being controlled at ~ 5 mTorr. RF power (150 W) was then applied to ignite the Ar plasma, which was directed towards the high-purity silver target to eject Ag atoms. After stabilization for 5 min, the shutter was opened, and the film started to grow on the rotating substrates.

### Fabrication of Ag_2_S-Based MCAs

The SiO_2_ (70 nm thick)/Si substrate was successively immersed in acetone, isopropanol and deionized water for surface cleaning. Afterwards, electron-beam lithography (EBL) was employed to pattern the bottom electrodes, and 50 nm-thick Au bottom electrodes were formed by thermal evaporation and lift-off processes. To pattern the electrolyte island at the designated cross-points, another EBL process was conducted. The Ag_2_S film was then deposited on pre-patterned substrate using the developed reactive sputter process. After lift-off, the Ag_2_S islands were formed at pre-defined cross-points. The top silver electrodes (50 nm-thick) were formed by conventional silver evaporation and lift-off processes. Finally, the crossbar arrays were annealed at different temperatures for 3 h.

### Characterization of Materials and Devices

The Ag_2_S film thickness was measured with a stylus profiler. Atomic force microscopy (AFM) was utilized for analyzing the film morphology. The chemical composition and phase structure of the films were determined using Rutherford backscattering spectrometry (RBS) and X-ray diffraction (XRD), respectively. Scanning electron microscope (SEM) images were collected with an ASO2 Zeiss 1550 scanning electron microscope, and energy-dispersive spectroscopy (EDS) was used to determine the composition of the silver clusters. Focused ion beam was utilized to extract the cross-section of films. All electrical characterization was conducted using Agilent B1500A Semiconductor Device Analyzer with waveform generator and fast measurement units.

### Demonstration of Multiply-Accumulate Calculation

The vertical and horizontal edge detection kernels were employed for image processing demonstration. The 3 × 3 kernel was encoded into the conductance of 18 memristors, with a single kernel value being represented by the conductance difference between two devices. For instance, the kernel value “-1” was encoded by the product of G_HRS_-G_LRS_, where G_HRS_ and G_LRS_ represent the conductance of high resistance state (HRS) and low resistance state (LRS), respectively. In analogue multiply-accumulate calculation, the positive reading bias was sent to the HRS and the negative one was sent to the LRS (the amplitude of positive/negative bias was the same, as transferred from the pixel value from the original image), resulting a negative net current to represent the negative output. Among 18 memristors, only 6 LRS devices are needed since there are 6 non-zero elements in the kernel array. Please note that these 6 LRS memristive units representing the kernel values of those "1" and "-1", dominate the overall output current in a multiply-accumulate calculation. While the units at HRS representing those "0" contribute negligible differential currents. This is illustrated by the significant difference between the encoded "1" (red-marked), "0" (green-marked) and "− 1" (blue-marked) in the vertical edge detection kernel (Fig. 4c). As a proof-of-concept demonstration, the dataset of multiply-accumulate calculations can be built by collecting the differential currents of these 6 LRS memristors (with input voltages), allowing the reference of corresponding output currents for any input voltages. The original image in our demonstration has 8-bit color depth, in which the pixel value ranges from 0 to 255. After kernel encoding, the pixel values of the original image were converted into read voltages with amplitudes ranging from 0 ~  ± 25.5 mV. This voltage range is both small enough to prevent any alteration in the kernel values and distinct enough to uniquely represent each pixel value. The linear *I*–*V* relationship of the LRS units across the whole input voltage window verifies the analogue multiply-accumulate calculation using Ohm's law and Kirchhoff's current law. In this demonstration, we read out all corresponding output currents for the pixels from this measured dataset, without physically sending all reading pulses to the MCA. After collecting all output currents, the pixel information can be decoded, and the convoluted image can be visualized.

## Results and Discussions

### CMOS-Compatible Fabrication of Wafer-Scale Ag_2_S-Based MCAs

The Ag_2_S thin films were deposited using reactive sputtering, as illustrated in Fig. S1a. A high purity silver target was sputtered in an atmosphere of Ar and H_2_S. Ag atoms were sputtered and reacted with H_2_S gas. The Ag_2_S thin films were deposited onto rotating substrates at room temperature, with a stable deposition rate of approximately 6 nm min^−1^ (Fig. S1b). The AFM 2D topographical map of a 50 nm Ag_2_S film deposited on a 4-inch SiO_2_/Si wafer shows an average surface roughness (*R*_a_) ranging from ~ 3 to ~ 4.5 nm at different locations (Fig. S2). The film composition was determined by RBS. The result (Fig. S3) displays robust signals corresponding to silver and sulfur respectively, with a Ag:S atomic ratio of approximately 2:1 obtained from the quantitative simulation. Post-annealing at 160 °C (for 3 h) was sufficient to obtain the monoclinic phase in a polycrystalline Ag_2_S film, as confirmed by XRD experiments (Fig. S4).

**Fig. 1:**
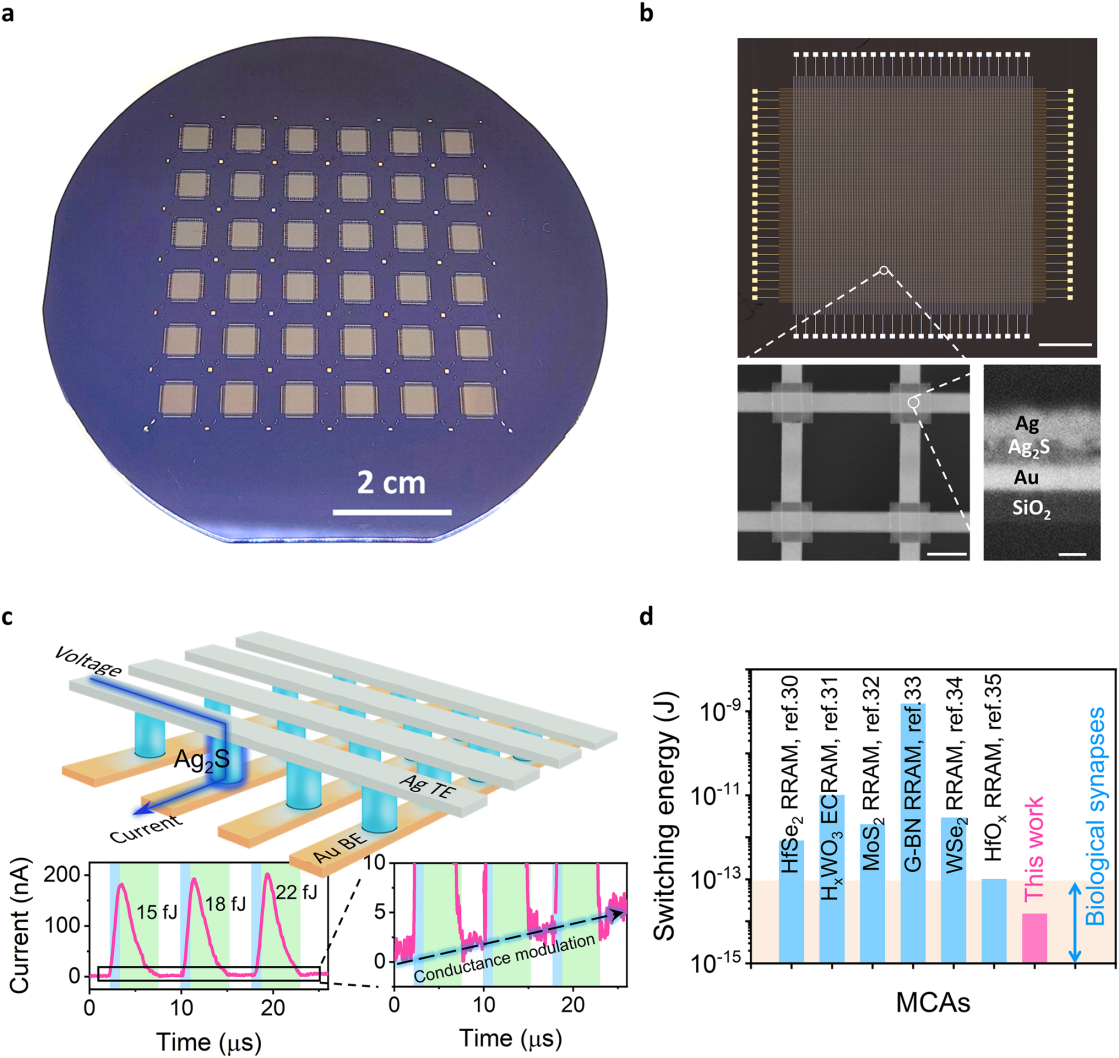
Wafer-scale Ag_2_S-based MCAs with ultra-low switching-energy. **a** An optical image of the Ag_2_S-based MCAs fabricated on a 4-inch SiO_2_/Si wafer. Scale bar: 2 cm. **b** An optical micrograph of a Ag_2_S-based MCA (top panel, scale bar: 1 mm), a top-view SEM image of a 2 × 2 sub-array (left-bottom panel, scale bar: 10 μm) and a cross-sectional SEM image of a memristive unit (right-bottom panel, scale bar: 50 nm). **c** Schematic illustration of the crossbar structure (top panel). A selected Ag_2_S device between a Ag TE line and a Au BE line was programmed by write (− 0.3 V, 1 μs)-read (5 mV) pulses, with current traces shown in left-bottom panel. The switching-energy of each writing pulse is calculated and marked. The light blue and light green shadings highlight the writing and the recovery time (after switching to reading pulse), respectively, and the reading regime in black dashed line is zoomed-in in the right-bottom panel. **d** Comparison of the switching-energy of the integrated Ag_2_S-based memristors and recently reported memristors in their crossbar arrays. The light orange shading marks the energy consumptions of biological synapses per synaptic event

The facile synthesis of Ag_2_S films at low temperature enables CMOS-compatible wafer-scale integration of Ag_2_S-based MCAs (Fig. 1a; the fabrication process is illustrated in Fig. S5 and detailed in the Experimental Section). An important feature of our array fabrication is the removal of excess Ag_2_S to eliminate additional sneak paths through the electrolyte. Compared with some reported 2D materials-based MCAs which employ universal electrolytes covering the entire array [23], our approach minimizes the crosstalk and interference between neighboring memristive units. The top panel of Fig. 1b presents an optical micrograph of a crossbar array, wherein the memristive units, each with a lateral dimension of 5 × 5 µm^2^, are situated at the cross-points between the top electrode (TE) and bottom electrode (BE). Further insights to a 2 × 2 sub-array and the sandwiched layer stack of a single Au/Ag_2_S (50 nm-thick)/Ag memristive unit are provided by the zoomed-in SEM images in the bottom panel of Fig. 1b. As illustrated in Fig. 1c, the selected memristive unit can be programmed by applying writing voltages between the corresponding columns and rows to modulate its conductance. We applied rapid write-read pulses to the Ag TE of the device (with a Au/Ag_2_S (200 nm thick, 160 °C-annealed)/Ag stack), and recorded the current response. Three consequent write pulses (− 0.3 V, 1 µs, light blue shaded at the left-bottom of Fig. 1c) gradually increase the device current during writing. A zoomed-in view of the reading process (5 mV, as shown at the right-bottom of Fig. 1c) clearly reveals the discrete conductive states. To calculate the switching-energy during device writing, we integrated the product of the writing voltage and device current during the entire duration of the writing pulses. The result shows that the memristive unit in the Ag_2_S MCA only consumes tens of femtojoule for each writing operation, which is comparable to the energy consumption per synaptic event of human synapses. The switching-energy of our device is also benchmarked with recently reported CMOS-compatible MCAs, showing the lowest switching-energy among these artificial synaptic devices (Fig. 1d; see the specific device properties in Table S1) [30–35]. Since both the device current and the parasitic capacitance are directly correlated to the lateral dimensions of the cross-points of the array, the energy efficiency and the operation speed of our Ag_2_S-based MCA could be further improved by device size downscaling (see discussions in Note S1).

### Efficient Silver Filament Formation

**Fig. 2:**
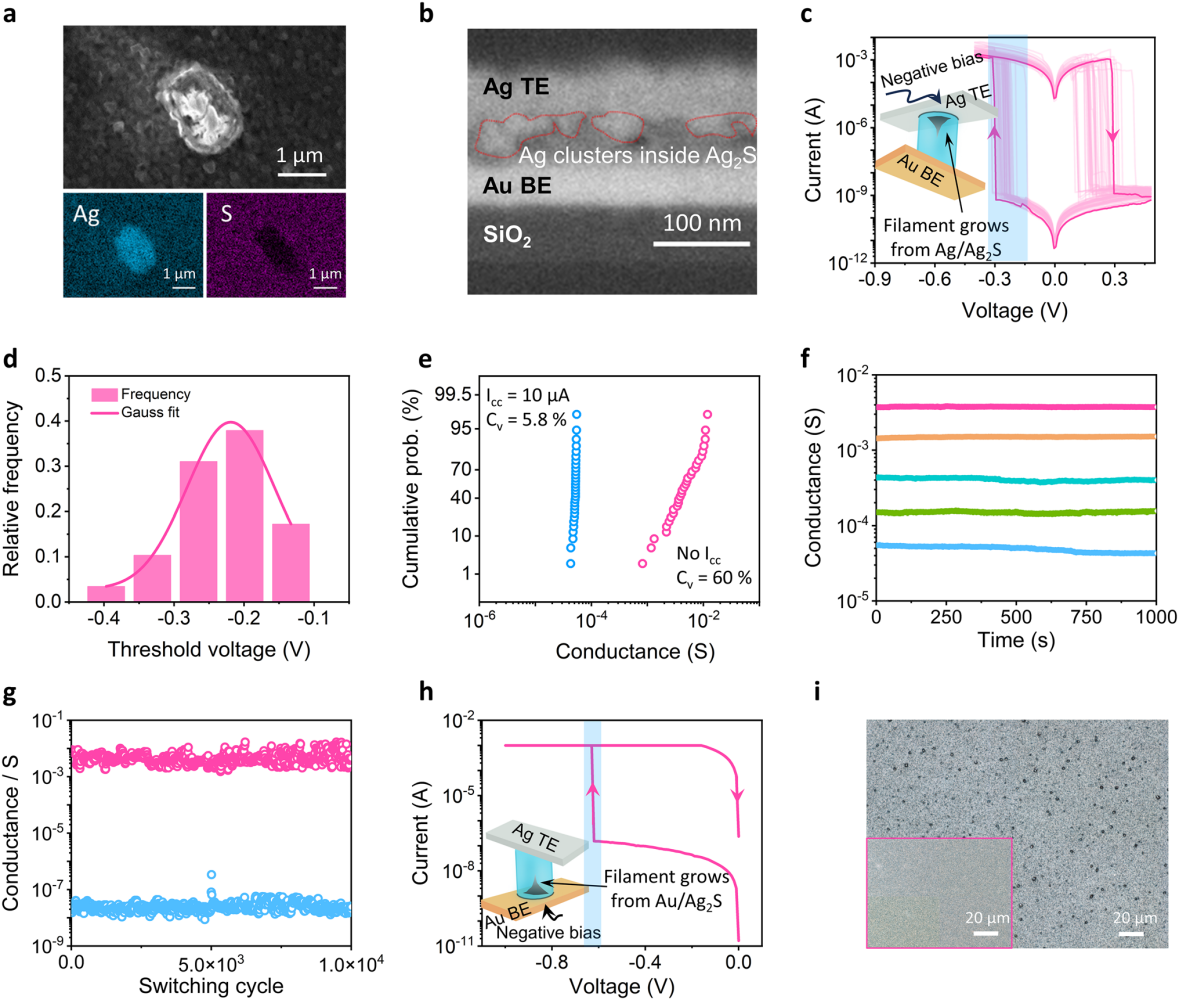
Efficient silver filament formation in Ag_2_S. **a** SEM image of the Ag_2_S film (top), with a formed silver cluster on the surface (using an accelerating voltage of 4.5 kV), and the corresponding EDS images (bottom-left for Ag signals and bottom-right for S signals). Scale bars: 1 μm. **b** Cross-sectional SEM image of an Au/Ag_2_S/Ag cell taken with 2 kV as the accelerating voltage. The red dash line highlights the electron-beam induced silver clusters inside the Ag_2_S layer. Scale bar: 100 nm. **c** *I–V* characteristics of the Au/Ag_2_S (50 nm)/Ag device under 50 cycles d.c. bias (applied to the Ag TE). The silver filament grows from Ag TE/Ag_2_S interface at V_th_ < − 0.3 V. **d** *V*_th_ distribution based on results from 30 devices and its Gauss fit. **e** Cumulative probability of the LRS conductance with or without *I*_cc_ (10 μA). The coefficient of variation (*C*_v_) was calculated. **f** Retention of conductance read under 5 mV bias. **g** Endurance of the device under 10^4^ switching cycles. The device was set or reset by − 0.3 V or 0.3 V pulses (10 ms) respectively, and the conductance was read at 5 mV after set/reset processes. **h** *I–V* characteristics of the Au/Ag_2_S/Ag device when 0 → − 1 → 0 V d.c. bias was applied to the Au BE. The silver filament grows from Au BE/Ag_2_S interface at *V*_th_ ≈ − 0.6 V. **i** Optical images of Ag_2+δ_S films sputtered on Ag, or Au (left bottom) substrates. The black dots are silver particles formed during the sputtering. Scale bars: 20 μm

The ultra-low switching-energy indicates efficient Ag^+^ ion reduction and Ag filament formation inside the Ag_2_S electrolyte. To confirm this, we conducted in-situ observations of the Ag^+^ ion reduction process in the Ag_2_S film upon electron beam scanning in a SEM instrument. The electron beam irradiation on the Ag_2_S during imaging could induce Ag^+^ migration toward and reduction on the film surface, leading to the rapid growth of silver clusters as shown in Fig. S6. Figure 2a shows a formed silver cluster. The stronger Ag signals along with the weaker S signals (in contrast to the surrounding Ag_2_S film) obtained during EDS mapping confirm the chemical composition of the identified particle. These silver clusters were also formed during the cross-sectional SEM analysis of an Au/Ag_2_S/Ag memristive cell, as highlighted by the dashed lines in Fig. 2b. This fast Ag^+^ migration and reduction inside Ag_2_S electrolytes results in an ultra-low threshold voltage (*V*_th_, the voltage applied to the Ag TE at which the abrupt current change is observed) for the silver filament formation. As shown in Fig. 2c, the memristor fabricated with 50 nm-thick Ag_2_S electrolyte shows *V*_th_ < − 0.3 V during consecutive d.c. operations. The *V*_th_ during set/reset processes exhibits cyclic variations (evaluated by coefficient of variation *C*_v_, calculated by dividing the standard deviation with population mean value) at 12.5% and 30.9% respectively, which is resulted from the stochastic dynamics of filament formation and ablation (see statistics in Fig. S7). The resulting LRS remains stable until a positive voltage is applied to reset the device back to its HRS, yielding a substantial dynamic range spanning 6 orders of magnitude. The statistical analysis of *V*_th_ based on random 30 devices exhibits a mean value of − 0.22 V based on a Gauss fit (Fig. 2d). The highest LRS conductance of our device could reach the milli siemens regime, with a device-to-device variation approaching 60% as summarized in Fig. 2e. This variation results from the inherent stochastic nature of the filament formation along the defects and grain boundaries inside the electrolyte [36–38], which can be suppressed by implementing current compliance (*I*_cc_) during d.c. settings (see the reduced *C*_v_ down to 5.8% with the *I*_cc_ of 10 μA in Fig. 2e. The results under different *I*_cc_ values are summarized in Fig. S8). Moreover, the retention test was conducted to examine the stability of these conductive states, showing the nonvolatile characteristics with multiple-level conductance tunability (Fig. 2f). The device was also subjected to repeated RS across the full dynamic range, revealing robust switching behavior over 10^4^ write-read cycles for endurance evaluations (Fig. 2g).

The *V*_th_ of our device is notably lower than those observed for previously reported CBMs, including state-of-the-art 2D material-based standalone memristors and their crossbar arrays [30, 39, 40]. Since the negative setting bias was applied to the Ag TE, silver filaments grew from the Ag TE/Ag_2_S interface during the setting process. Conversely, we show that setting the same device on Au/Ag_2_S interface (by applying negative bias to the Au BE) requires a much higher *V*_th_ (Fig. 2h), which indicates a preference for silver nucleation at the Ag/Ag_2_S interface over the Au/Ag_2_S interface. Indeed, silver nucleation on the Au surface results in a new Au/Ag interface which creates an extra kinetic energy barrier for the process to overcome. To further verify this finding, we investigated distinct silver nucleation behaviors by sputtering Ag_2+δ_S films on Ag or Au substrates, respectively. The excess Ag^+^ ions from the silver-rich Ag_2+δ_S can indeed form silver particles on Ag substrates (the black dots in Fig. 2i) during sputtering. While the nucleation was suppressed by the higher barrier on the Au surface (see left bottom of Fig. 2i). In common CBMs (such as those based on a-Si:Ag, HfSe_2_:Ti and PdSe_2_:Ti material systems), electrolytes cannot provide sufficient mobile metal ions for filament formation. These ions must be provided by oxidizing an active electrode (anode) during the setting process, and then migrate to the cathode (which is usually an inert metal electrode with a high nucleation barrier) under electric fields for subsequent filament growth [30, 40, 41]. In direct contrast, Ag_2_S electrolyte can act as a Ag^+^ reservoir, directly supplying mobile Ag^+^ for filament formation. The low nucleation barrier at the Ag/Ag_2_S interface together with the Ag^+^ supply from the solid-electrolyte leads to the low *V*_th_ value observed in our Ag_2_S-based memristors.

### Threshold Reduction by Enhancing Ag^+^ Migration

**Fig. 3:**
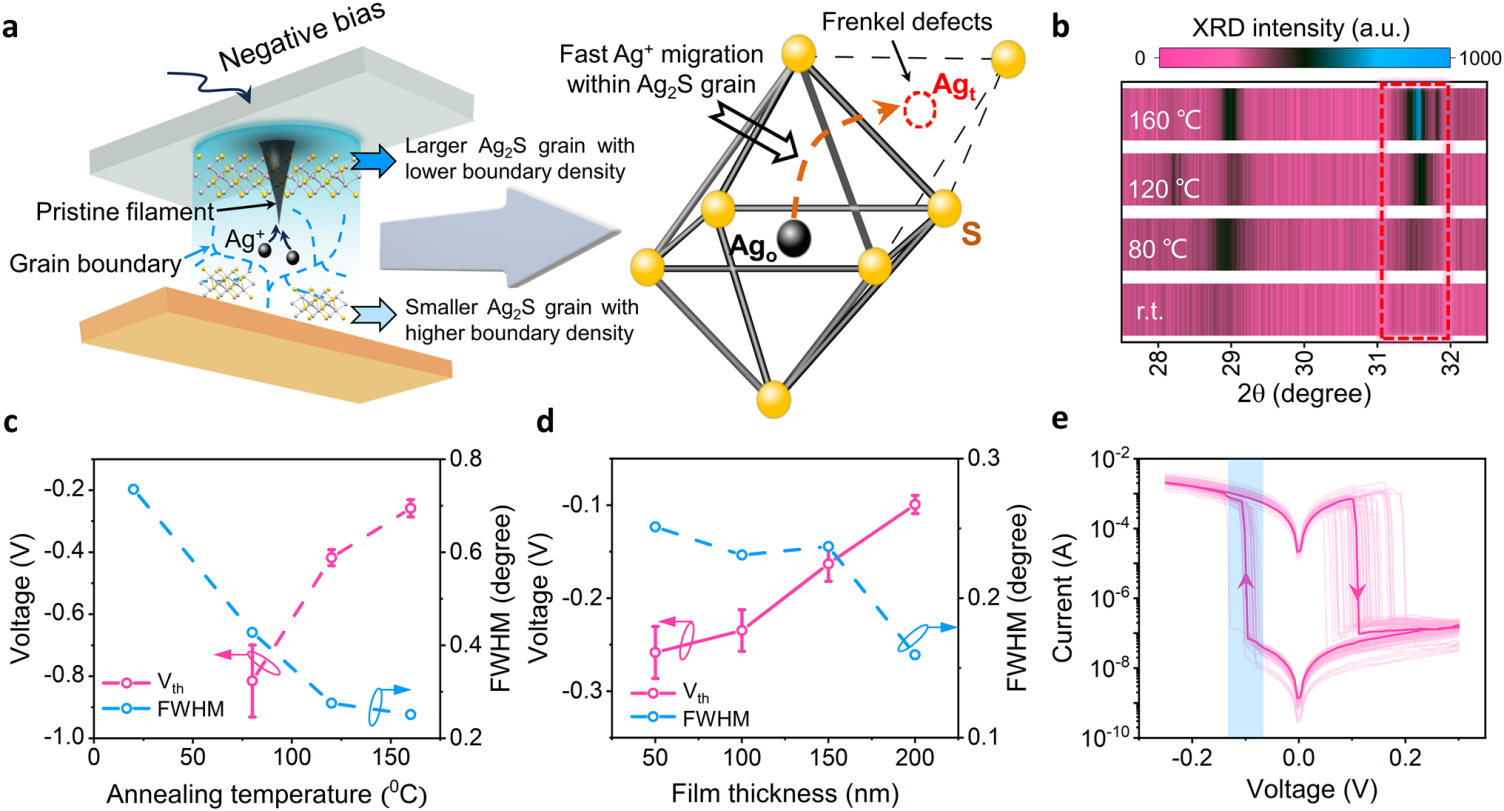
Enhancing Ag^+^ migration to further decrease *V*_th_. **a** Schematic illustration of filament growth inside the Ag_2_S electrolyte and Ag^+^ migration within the Ag_2_S lattice. The fast Ag^+^ migration through Frenkel defects (such as octahedral (Ag_o_) and tetrahedral (Ag_t_) sites) inside large Ag_2_S grains facilitates the formation of pristine filaments at top Ag_2_S layers, which enables the low *V*_th_. **b** XRD diffractogram of 50 nm-thick Ag_2_S films annealed at different temperatures. **c** *V*_th_ of the Ag_2_S (50 nm)-based memristors fabricated with different annealing temperatures. The FWHM values of different films were simulated based on the diffraction singlet at 2θ ≈ 31.5°. **d** *V*_th_ of the Ag_2_S (160 °C annealed)-based memristors fabricated using Ag_2_S films with different thickness. The FWHM values of different films were simulated based on the diffraction singlet at 2θ ≈ 31.5°. **e** 50 cycles *I*–*V* characteristics of the device with 200 nm Ag_2_S films (160 °C-annealed)

With the low kinetic energy barrier of Ag nucleation at the Ag/Ag_2_S cathode interface, enhancing Ag^+^ migration inside the Ag_2_S electrolyte could potentially further reduce the required energy input (thus *V*_th_) to form silver filaments. Previous material studies have reported that monoclinic Ag_2_S offers substantial Frenkel defects in its grain lattice. These allow Ag^+^ ions to move between adjacent octahedral and tetrahedral sites with a low activation energy (~ 0.2 eV), facilitating faster ion migration through the crystal lattice over grain boundaries under electric fields (as illustrated in Fig. 3a) [42, 43]. To explore the impact of Ag^+^ migration on filament formation, we annealed the deposited Ag_2_S films at different temperatures (up to 160 °C, below the transition temperature from monoclinic structure to body-centered cubic structure). The XRD diffractogram (Fig. 3b, c) clearly shows stronger diffraction intensity and smaller full width at half maximum (FWHM) for films with higher annealing temperatures. The decrease in FWHM signifies an increase of Ag_2_S grain size, promoting a long-range ordered lattice structure that decreases the energy barrier for Ag^+^ ion migration within Ag_2_S electrolytes. Indeed, our devices fabricated with higher post-annealing temperatures exhibit lower V_th_ values (see Figs. 3c and S9), and no RS could be observed in a non-annealed device with a bias under ± 1 V (Fig. S10).

Moreover, XRD analysis reveals that the FWHM further decreases in thicker films (from 50 to 200 nm, after 160 °C post annealing), indicating the presence of larger grains in the top layers of the sputtered thick Ag_2_S films (Figs. 3d and S11). This observation is consistent with the guide effect from atomic orientation of substrates during film deposition [44, 45]. Initially, Ag_2_S deposited on the amorphous substrate starts with poorly ordered structures due to the absence of crystalline templates. As the film grows thicker, subsequent layers of Ag_2_S are deposited on already formed Ag_2_S rather than directly on the amorphous substrate. The poorly ordered region serves as nucleation sites for further crystallization, leading to the development of a polycrystalline Ag_2_S structure. Consequently, the bottom side of the deposited Ag_2_S layer has a relatively disordered structure, while the top layers exhibit larger grains with lower boundary density (Fig. S11). For thick Ag_2_S film with highly crystallized structure at the top Ag/Ag_2_S interface, Ag^+^ migration towards the Ag TE is easier, leading to the more energy-efficient filament formation compared to that occurring in the thin Ag_2_S film with poor crystalline surface. Although subsequent filament growth extends to the bottom Ag_2_S layers, the relatively inefficient Ag^+^ migration in the poorly crystallized bottom Ag_2_S layers can be compensated by the self-promoting effect of the pristine filaments: The sharp front of pristine Ag filament can promote the subsequential Ag^+^ supply by enhancing the local electric fields. The kinetics of the silver filament formation greatly resemble that of the dendrite growth observed in lithium ion batteries [46, 47]. Indeed, V_th_ for silver filament formation in our memristors decreases with thicker Ag_2_S films (Figs. 3d and S9). Remarkably, the Au/Ag_2_S (200 nm, 160 °C annealed)/Ag device exhibits V_th_ of about − 0.1 V under consecutive d.c. operations (Fig. 3e), which is the record low value among wafer-scale CMOS-compatible MCAs. This small V_th_ allows the device to operate under fast pulses, achieving the ultra-low switching-energy depicted in Fig. 1c. Further enhancing the local Ag^+^ migration by increasing the thickness of Ag_2_S electrolytes does not significantly decrease the *V*_th_ (Fig. S12), which may be limited by the required overpotential for the Ag^+^ reduction. Moreover, the statistical analysis of 30 Au/Ag_2_S (200 nm, 160 °C annealed)/Ag devices demonstrates the consistency of device behavior among the array, despite some device-to-device variations on *V*_th_ (Fig. S13).

### Computing Demonstration Using Flexible Ag_2_S-Based MCA

**Fig. 4:**
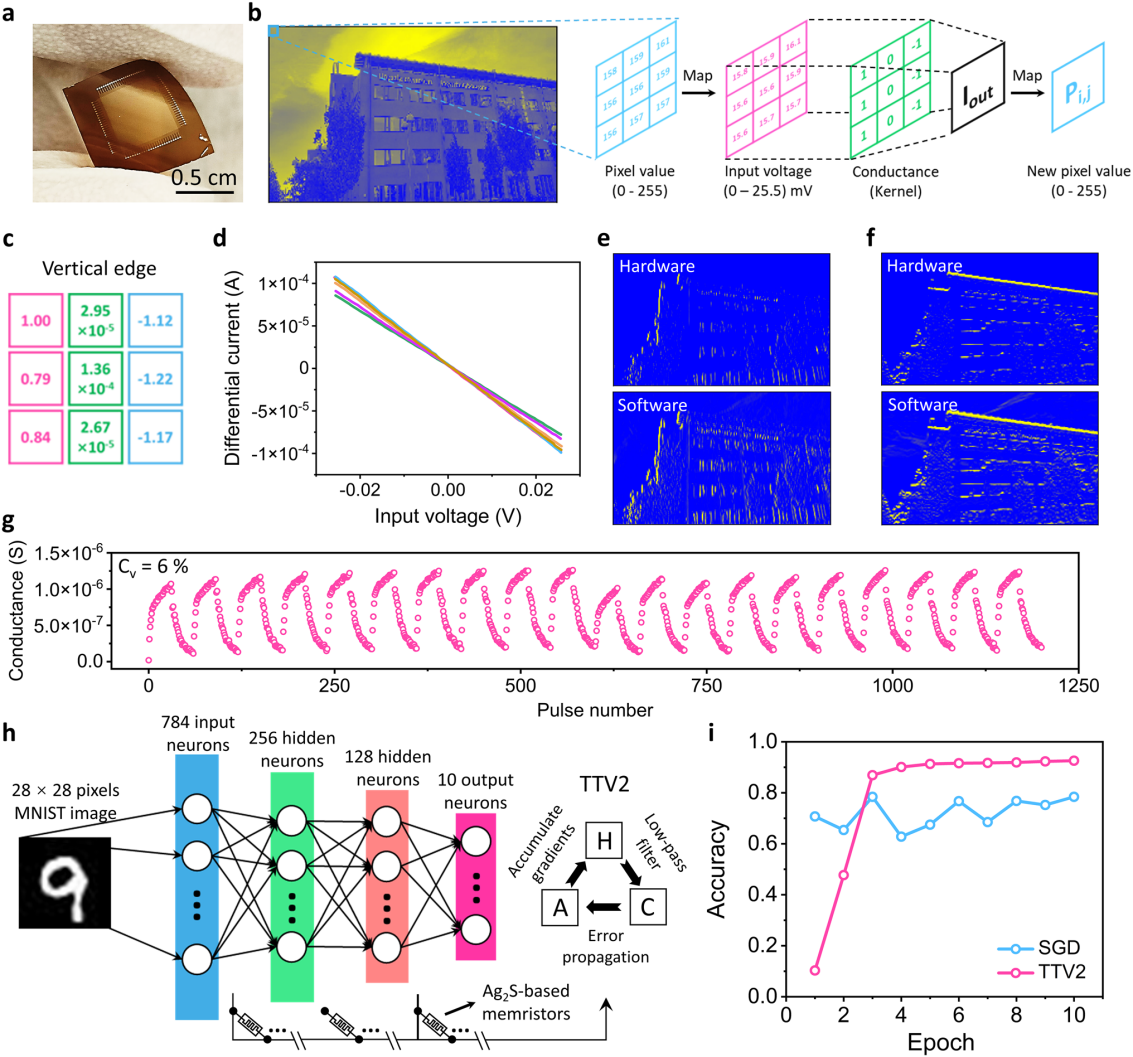
Demonstration of neuromorphic computing using the Ag_2_S-based flexible MCA. **a** Optical photograph of an MCA fabricated on polyimide substrate. **b** Illustration of image processing on the MCA. **c** Analogue kernel for vertical edge detection, which is normalized based on the encoded conductance using a (1,1) memristor as a reference (the conductance of this memristor is referred to as 1). Red, green, and blue-marked values represent “1”, “0” and “− 1” in the original digital kernel. **d** The differential currents of 6 LRS memristors, whose conductance represents the kernel values of those “1” and “− 1”. **e** Output images from hardware and software processing after vertical edge detection. **f** Output images from hardware and software processing after horizontal edge detection. **g** Programming of the Ag_2_S-based flexible memristors by consequent 30 potentiation pulses (− 0.3 V, 1 μs) and 30 depression pulses (0.3 V, 1 μs). A C_v_ of 6% was calculated from the maximum conductance during 1200-step programming to evaluate the cycle-to-cycle variation. **h** Schematic illustration of the DNN architecture. The conventional SGD and TTV2 are utilized as training algorithms, respectively. In TTV2, the weights are stored in two matrices (**a** and **c**), and matrix H serves as a low-pass filter to minimize the random noise from the inputs. **i** Simulated accuracy of DNN with the Ag_2_S-based memristors using SGD or TTV2 as the training algorithm

The low-temperature (≤ 160 °C) fabrication process also makes our Ag_2_S-based MCA very promising for applications in flexible electronics. To demonstrate this capability, we fabricated the same selector-less MCA on polyimide substrates (Fig. 4a). Thanks to the intrinsic ductility of Ag_2_S materials [24], the fabricated flexible MCA exhibits reliable switching behavior under bending with 3 mm radius (Fig. S14). We further conducted proof-of-concept demonstration of multiply-accumulate calculations on the flexible array. As illustrated in Fig. 4b (see details in Experimental Section), convolutional kernels were encoded into memristor conductance, and pixel values (ranging from 0 to 255) of the input image were mapped to the voltage of read bias (ranging from 0 to 25.5 mV). Feeding read bias to different rows generated an overall current in the shared column, which was weighted from the encoded conductance. After performing the convolution process along the entire input image, an output image can be generated. The encoded vertical edge detection kernel is shown in Fig. 4c, with 6 LRS memristive units representing the kernel values of those "1" and "− 1". Under bending with 3 mm radius, we fed the reading voltages (0 ~  ± 25.5 mV) to these 6 LRS memristive units and collected their differential currents across the whole input voltage window (Fig. 4d), which was utilized to establish a dataset of multiply-accumulate calculations. The corresponding output currents from any input voltages were referenced from the dataset for subsequent decoding. The output images from hardware processing and software simulation, after vertical (Fig. 4e) and horizontal (Fig. 4f) edge detection, demonstrate the promising computing capabilities of our Ag_2_S-based MCA.

We also simulated the image classification using Modified National Institute of Standards and Technology (MNIST) dataset. As depicted in Fig. 4g, programming a memristive unit (under 3 mm bending radius) was conducted through 1200 up-and-down pulses (± 0.3 V, 1 µs) for weight potentiation and depression. The non-ideal switching characteristics were modeled (Fig. S15) to simulate the behavior of synaptic devices in a 4-layer DNN (Fig. 4h). After training 10 epochs with conventional stochastic gradient descent (SGD) algorithm, the DNN reaches a recognition accuracy of 78.4% on the testing dataset, which is lower than that of 93.2% from ideal devices (without non-linearity and asymmetry). To mitigate the influence of inherent non-ideality, we employed a specialized in-memory SGD training algorithm (referred as TTV2) [18, 19]. The advanced DNN with the TTV2 algorithm utilizes two matrices (A and C) to store weight information, and adds a low pass filter (H) between them (Fig. 4h). By utilizing the symmetry point shifting technique (see details in Fig. S16 and Note S2) and the low pass filter, the randomness from input data (e.g., unintended features, which act as short-term noise) can be significantly reduced, while the true features are effectively propagated to matrix C to sparsely update its weight values. Consequently, the accuracy of our Ag_2_S-based DNN reaches a remarkable saturation value of 92.6%, despite the inherent non-idealities of the memristive units.

## Conclusions

In conclusion, we demonstrate wafer-scale integration of Ag_2_S-based MCA with fully CMOS-compatible processes and a thermal budget under 160 °C. The low Ag nucleation barrier along with the improved Ag^+^ migration in Ag_2_S electrolytes enable a record-low *V*_th_ under d.c. operation for wafer-scale CMOS-compatible MCA. As a result, the integrated memristors exhibit an ultra-low switching-energy at femtojoules under sub-megahertz pulse operation, which is comparable to those of biological synapses. We further demonstrate that the Ag_2_S-based MCA can be fabricated on polyimide substrate for flexible applications. The flexible MCA achieves analogue multiply accumulate calculations for image processing, and demonstrates an impressive image recognition accuracy of 92.6% in DNN simulation. Therefore, our Ag_2_S-based MCAs hold great promise for energy-efficient neuromorphic computing.

## Supplementary Information

Below is the link to the electronic supplementary material.

Supplementary file1 (DOCX 17950 kb)
