# Supplementary material for: Wafer-Scale Ag2S-Based Memristive Crossbar Arrays with Ultra-Low Switching-Energies Reaching Biological Synapses
Source: Nanomicro Lett. 2024 Nov 22;17:69. doi: 10.1007/s40820-024-01559-2 (PMC11582288; doi:10.1007/s40820-024-01559-2)
Supplement: Supplementary file 1 — Supplementary file1 (DOCX 17950 kb) [file 40820_2024_1559_MOESM1_ESM.docx]

Supporting Information for

**Wafer-Scale Ag_2_S-Based Memristive Crossbar Arrays with Ultra-Low Switching-Energies Reaching Biological Synapses**

Yuan Zhu^1^, Tomas Nyberg^1^, Leif Nyholm^2^, Daniel Primetzhofer^3^, Xun Shi^4^ and Zhen Zhang^1,^*

^1^Division of Solid-State Electronics, Department of Electrical Engineering, Uppsala University, Uppsala 75121, Sweden

^2^Department of Chemistry, Uppsala University, Uppsala, Sweden

^3^Department of Physics and Astronomy, Uppsala University, Uppsala, Sweden

^4^State Key Laboratory of High Performance Ceramics and Superfine Microstructure, Shanghai Institute of Ceramics, Chinese Academy of Sciences, Shanghai 200050, P. R. China

*Corresponding author. E-mail: [zhen.zhang@angstrom.uu.se](mailto:zhen.zhang@angstrom.uu.se) (Zhen Zhang)

**Note S1 Operation speed of Ag_2_S-based memristive crossbar arrays (MCAs)**

The operation speed of memristors is limited by both the response time to the writing pulses, and the recovery time when switching to the reading pulses. The recovery time determines the time required to reliably read the conductance. As shown in Fig. 1c in the main text, during the transition from the write pulse to the read pulse, there is a current recovery process marked in light green shadings. This transient behavior is associated with the resistor-capacitor delay [S1]. The parasitic capacitance of memristor starts to charge/discharge due to the voltage switching. After complete charge transfer, the conductive states reach their steady value. Our device exhibits a recovery time of about 5 µs, allowing a write–read operation frequency approaching sub-megahertz range. Since both the setting current and the parasitic capacitance are directly correlated to the lateral dimensions of the cross-points of the array, the energy efficiency and the programming speed of our Ag_2_S-based MCA could be further improved by device downscaling.

**Note S2 Deep learning using advanced training algorithm to compensate for the non-idealities of the memristors**

In practical memristor-based hardware, the weight update is typically nonideal as also demonstrated for our Ag_2_S-based memristors (Fig. S15). With this characteristic, the conductance changes during potentiation and depression are different, and are typically functions of the present conductance. Under an up-and-down pulse train, the conductance would evolve to a stabilized value, denoted as the symmetric point, as demonstrated in Fig. S16). Specifically, if the device is at the high resistance state (HRS), it updates more weight in potentiation than that in depression, and vice versa. The conductance would thus be pushed towards the symmetric point under an up-and-down pulse train, regardless of the initial conductance. Finally, memristors at the symmetric point exhibit the same response to positive and negative programming pulses.

In deep learning neuro network (DNN) training, conventional stochastic gradient descent (SGD) and backpropagation algorithms update the weights to their optimized values for specific tasks. This is achieved by extracting the true feature from sufficient input data examples under supervised learning. However, the randomness (or unintended feature) from the input data is also introduced into the training process, which can trigger writing pulses to update synaptic weights. Considering the massive input examples in training datasets, all kinds of randomness could induce almost the same amount of positive and negative programming pulses, which serves the same function of consecutive up-and-down pulse train. As a result, the true feature updates the weight to their optimized value, while the random noise pushes the weight towards their symmetric points. The competition between them can significantly degrade the accuracy of DNNs.

In the advanced training algorithm TTV2, the weight information is stored in two matrices (A and C). The memristors in A are pre-set at their symmetric points, and they exhibit the same response to positive and negative writing pulses. With this symmetry point shifting technique, the randomness can be significantly filtered, and the elements in A represent the accumulated gradient information. TTV2 utilizes a low-pass function (H) to correlate the weight updates for A and C. When the accumulated weight in A exceeds the threshold (a manually set hyper-parameter), the corresponding value is reset back to the symmetry point, and a pulse train is applied to achieve a parallel update in C. In this process, the symmetric point is retained in A while the true feature is successfully learnt by C. Consequently, TTV2 can compensate the non-idealities of practical memristive hardware to achieve high computing accuracy [S2, S3].

**Supplementary Figures and Tables**


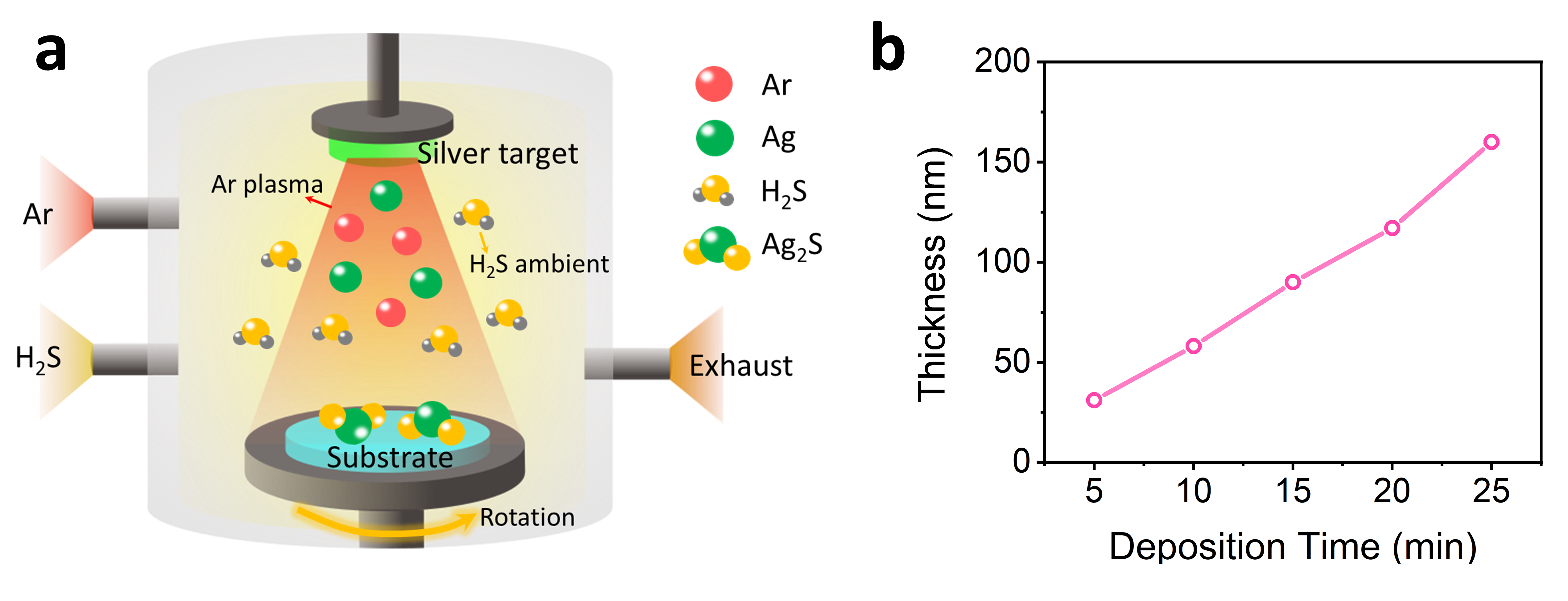


**Fig. S1** Synthesis of Ag_2_S by reactive sputtering. **a** Schematic illustration of the reactive sputter process. **b** The measured Ag_2_S film thickness as a function of the sputtering time. The results suggest a deposition rate of ~6 nm/min


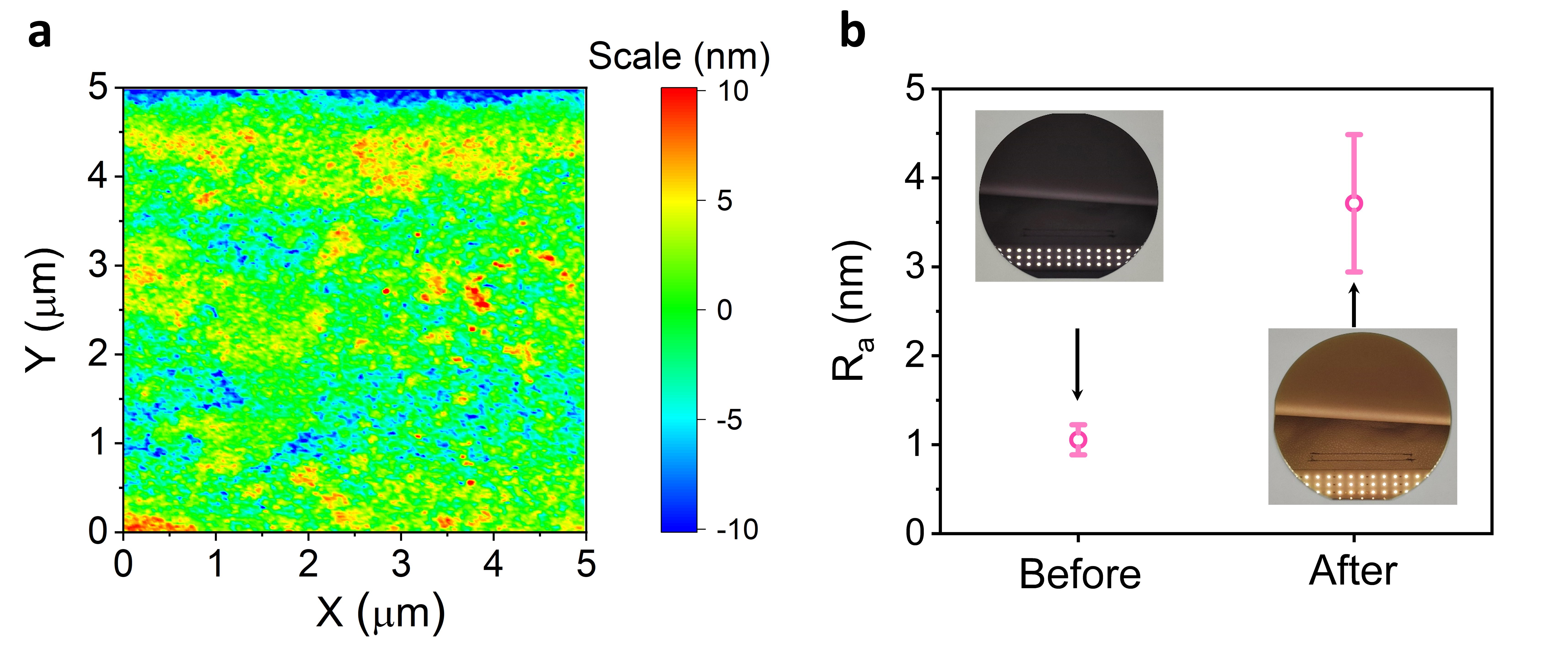


**Fig. S2** Surface roughness of the obtained Ag_2_S films. **a** The surface morphology of a 50 nm thick Ag_2_S film studied with atomic force microscopy. **b** The average surface roughness of the 4-inch wafer before and after deposition of a 50 nm thick Ag_2_S film. The insets show optical images of the wafer


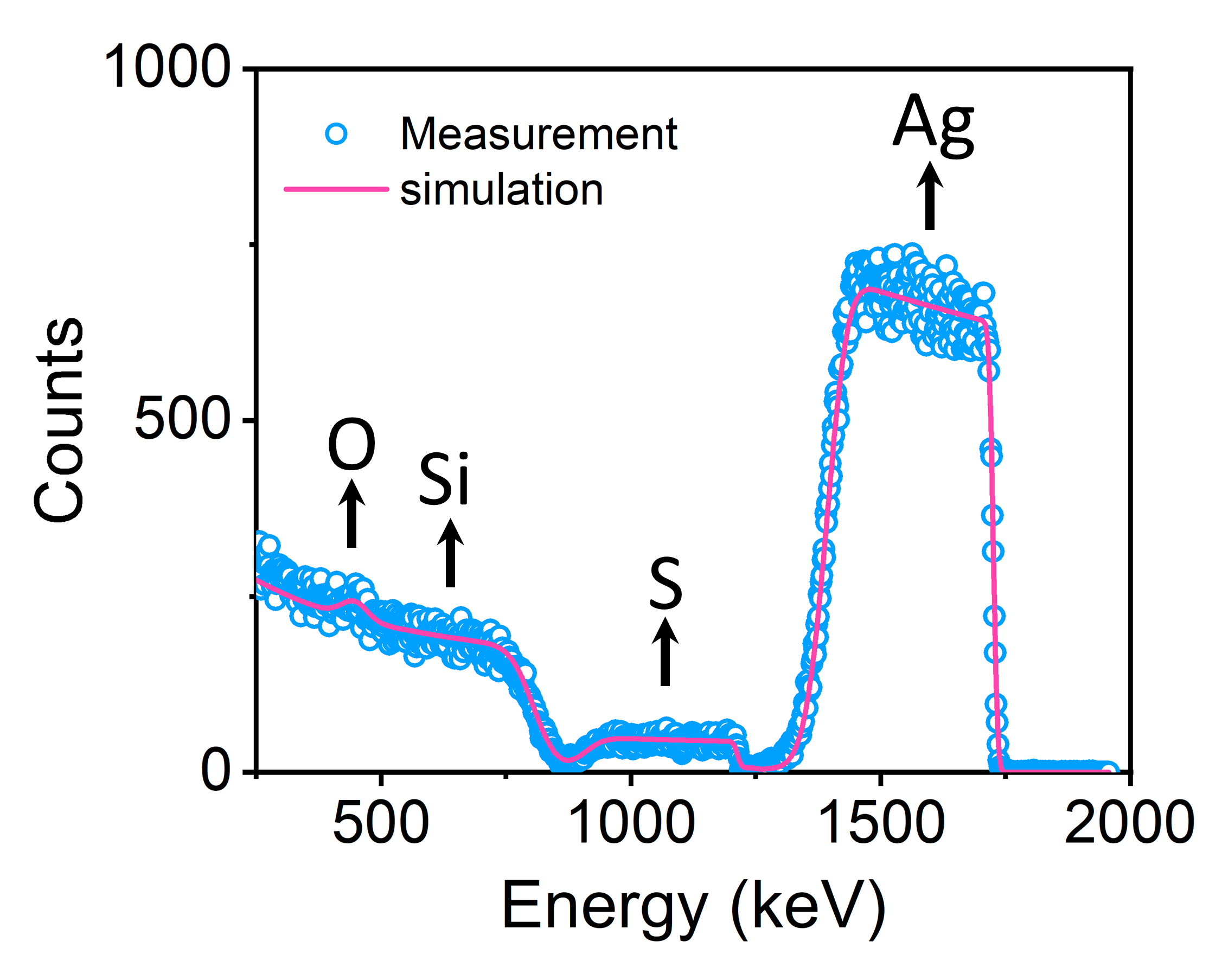


**Fig. S3** Rutherford backscattering spectrometry results for a 50 nm thick Ag_2_S film grown on a SiO_2_/Si substrate. A quantitative simulation yields an Ag:S atomic ratio of 0.665:0.335, with the silicon and oxygen signals stemming from the SiO_2_ substrate


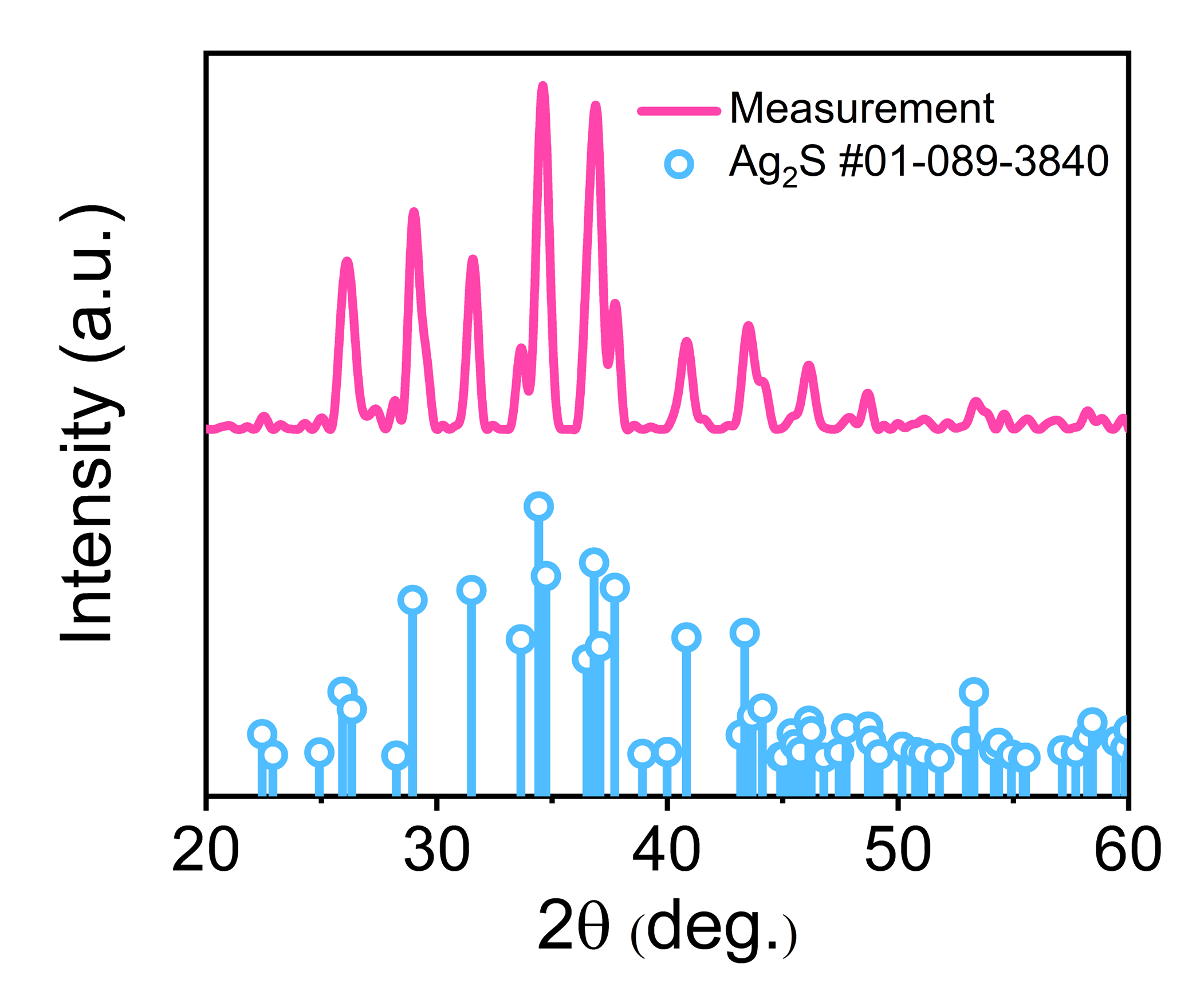


**Fig. S4** Phase structure of the obtained Ag_2_S film. X-ray diffractogram for the Ag_2_S film (annealed at 160 ^o^C) is consistent with that of the monoclinic Ag_2_S crystal (PDF#01-089-3840).


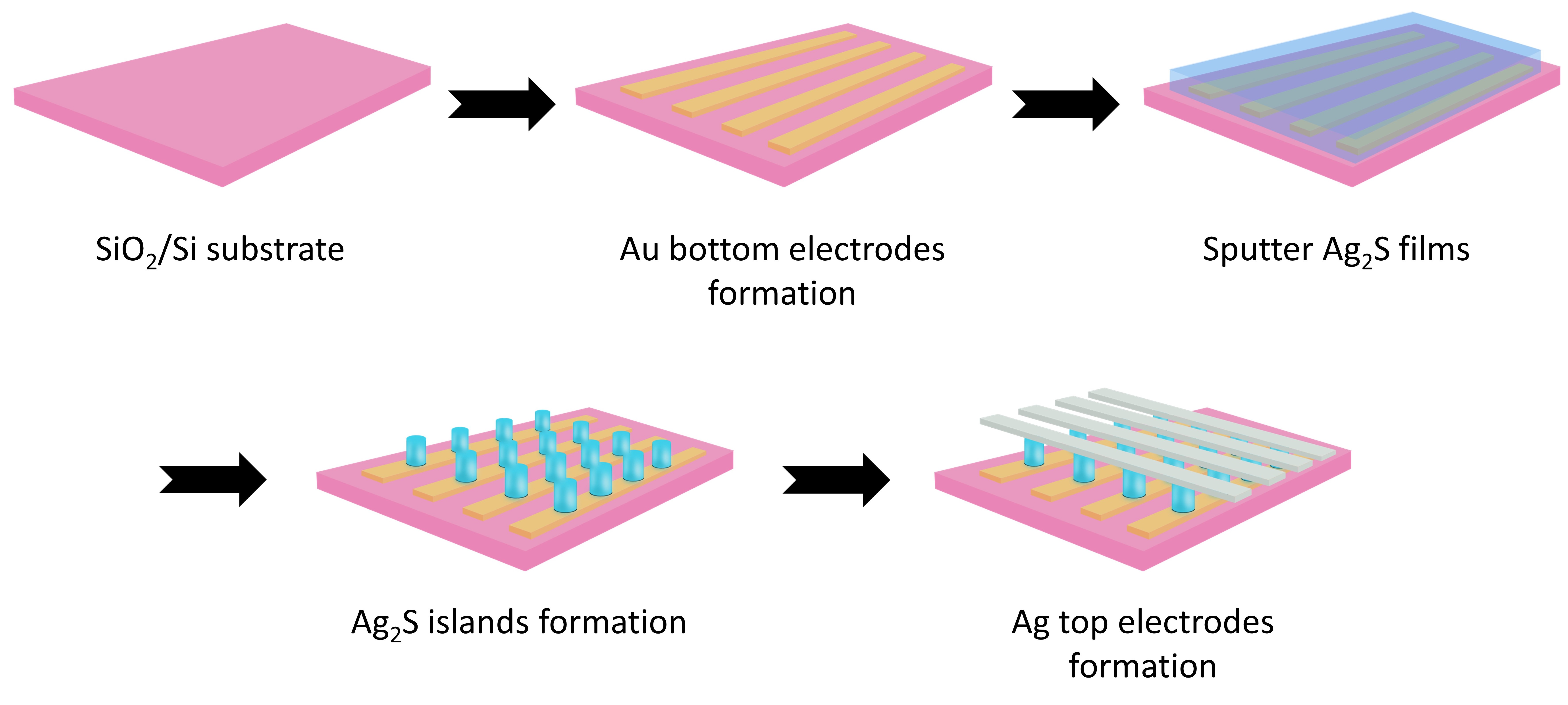


**Fig. S5** Ag_2_S-based MCA fabrication. Schematic illustration of the fabrication flow of the Ag_2_S-based memristive crossbar array. A pre-cleaned 4-inch SiO_2_ / Si wafer was utilized as the substrate. Electron-beam lithography (EBL) was employed to pattern the bottom electrode (BE) lines, followed by gold evaporation and lift-off processes to form 50 nm-thick Au BE. Afterwards, another EBL process was used to pattern the electrolyte island. The Ag_2_S film was then deposited on pre-patterned substrate using the developed reactive sputter process. Ag_2_S islands were formed at pre-defined cross-points after lift-off. The silver top electrodes (TE), also 50 nm-thick, were formed by silver evaporation and lift-off processes. Finally, the crossbar arrays were annealed at different temperatures (up to 160 ^o^C) for 3 hours.


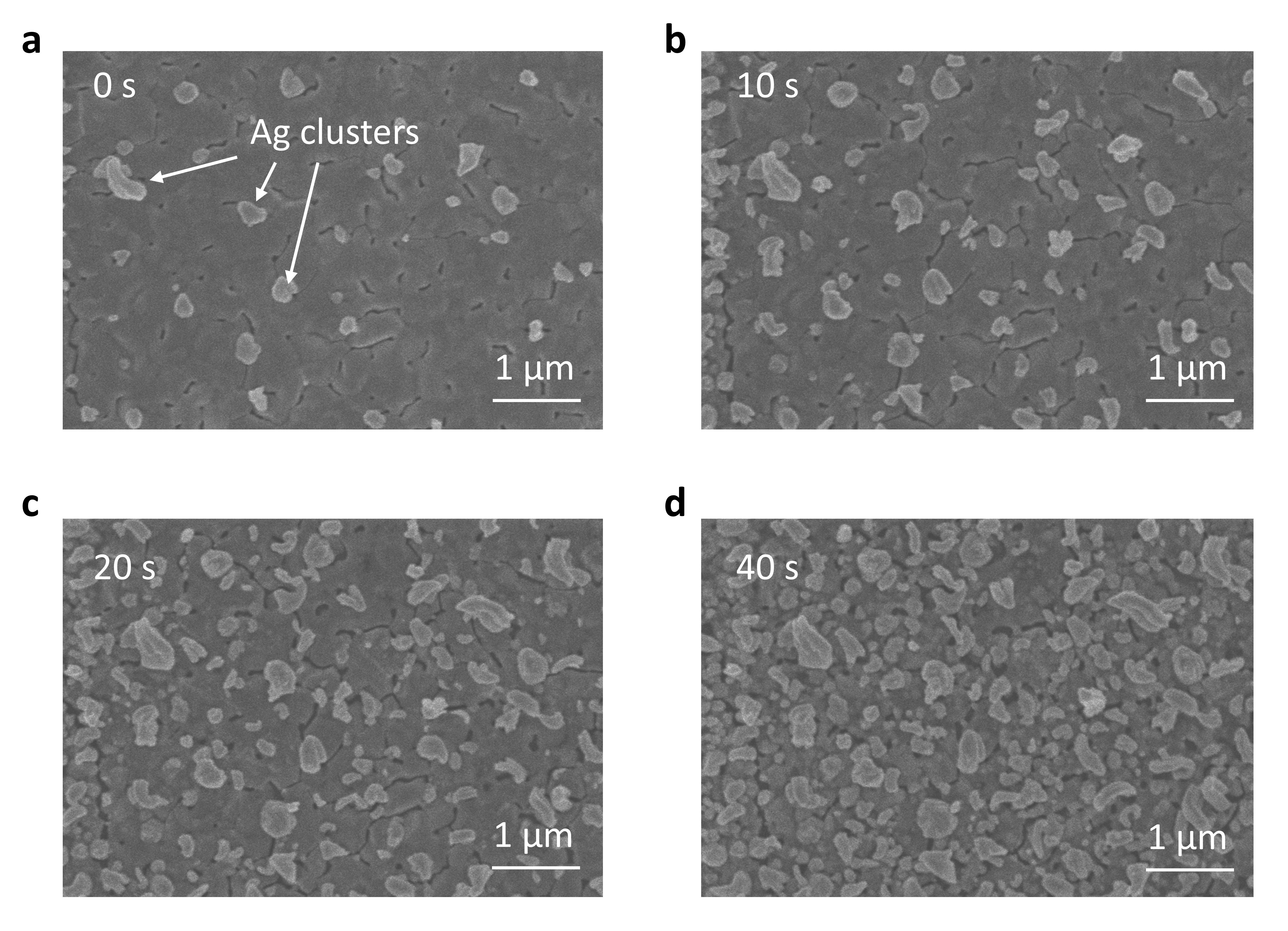


**Fig. S6** Observation of Ag^+^ ion migration and reduction under SEM. Scanning electron microscopy images of an Ag_2_S film for different electron-beam exposure times (**a**, 0 s; **b**, 10 s; **c**, 20 s; **d**, 40 s). The accelerating voltage was 4.5 kV. The silver clusters seen at 0 s (a) were formed during the focusing process. No silver clusters were observed on a raw film surface.


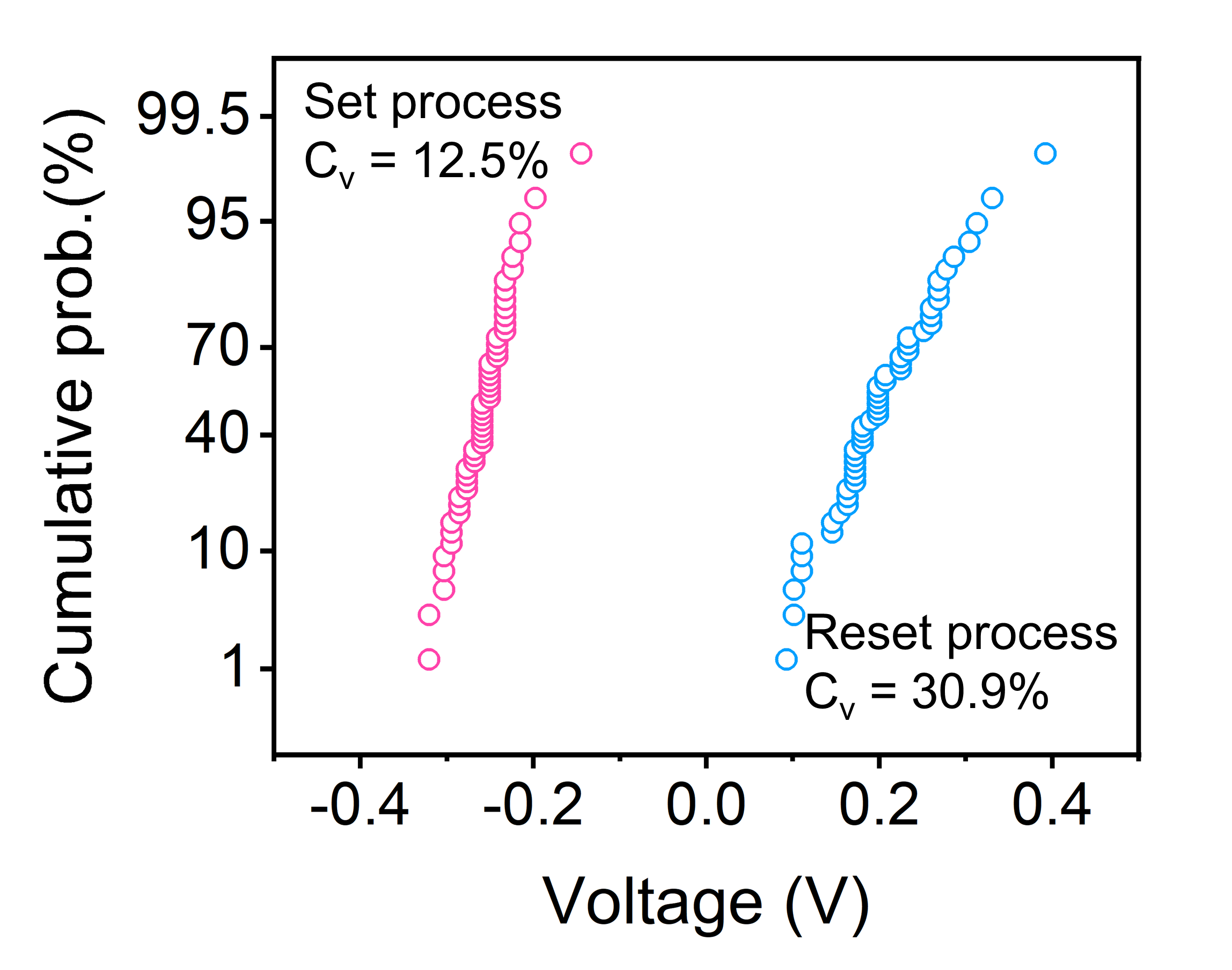


**Fig. S7** Cumulative probability of V_th_ of the Au/Ag_2_S (50 nm)/Ag device during consecutive set and reset processes. The corresponding I-V characteristics can be found in Fig. 2c


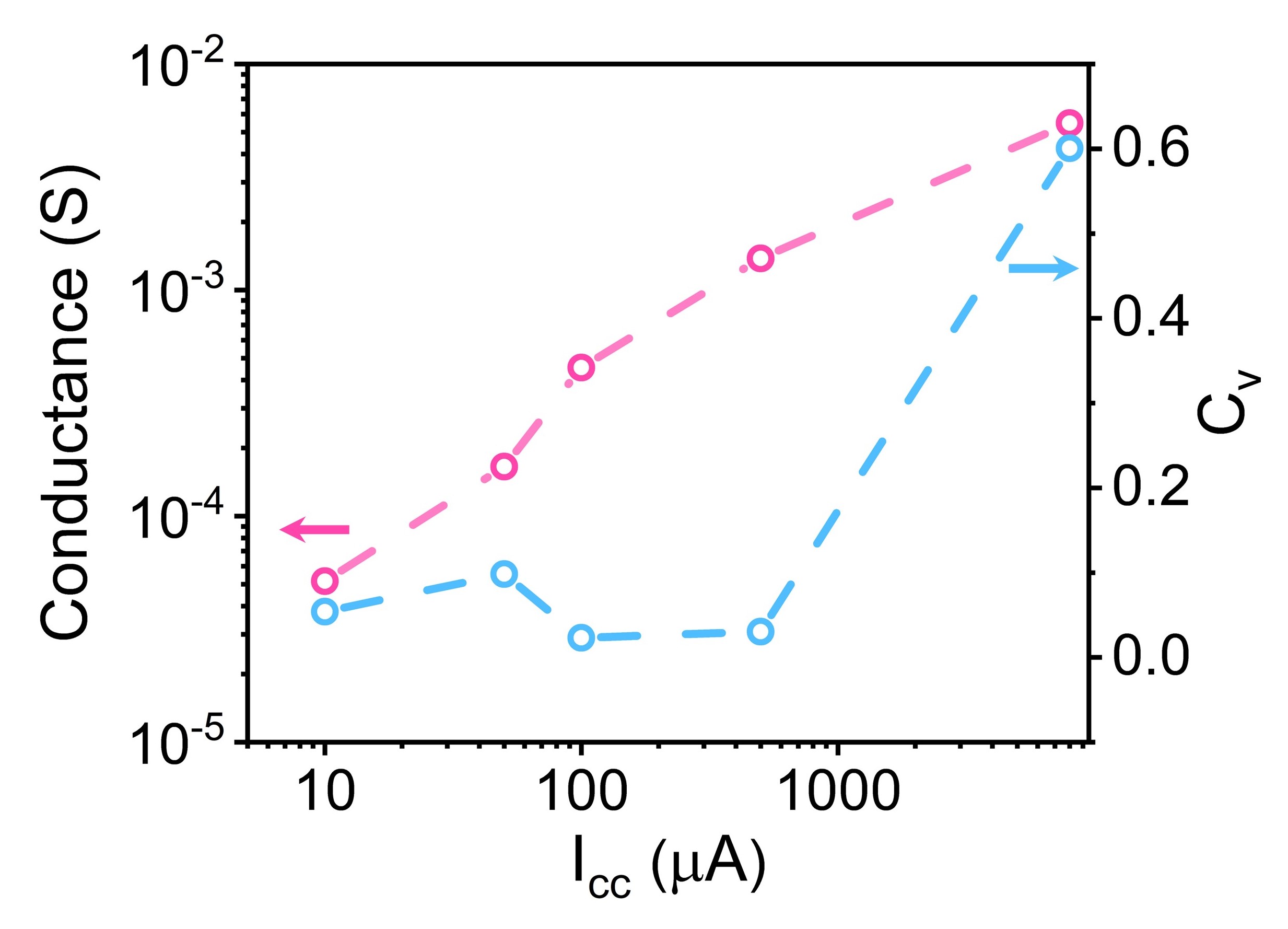


**Fig. S8** Suppressing the stochasticity of the filament formation by current compliance. The mean conductance value (red-marked) and their coefficient of variation (blue-marked) under different current compliance (i.e., 10 μA, 50 μA, 100 μA and 500 μA) during d.c. settings were summarized


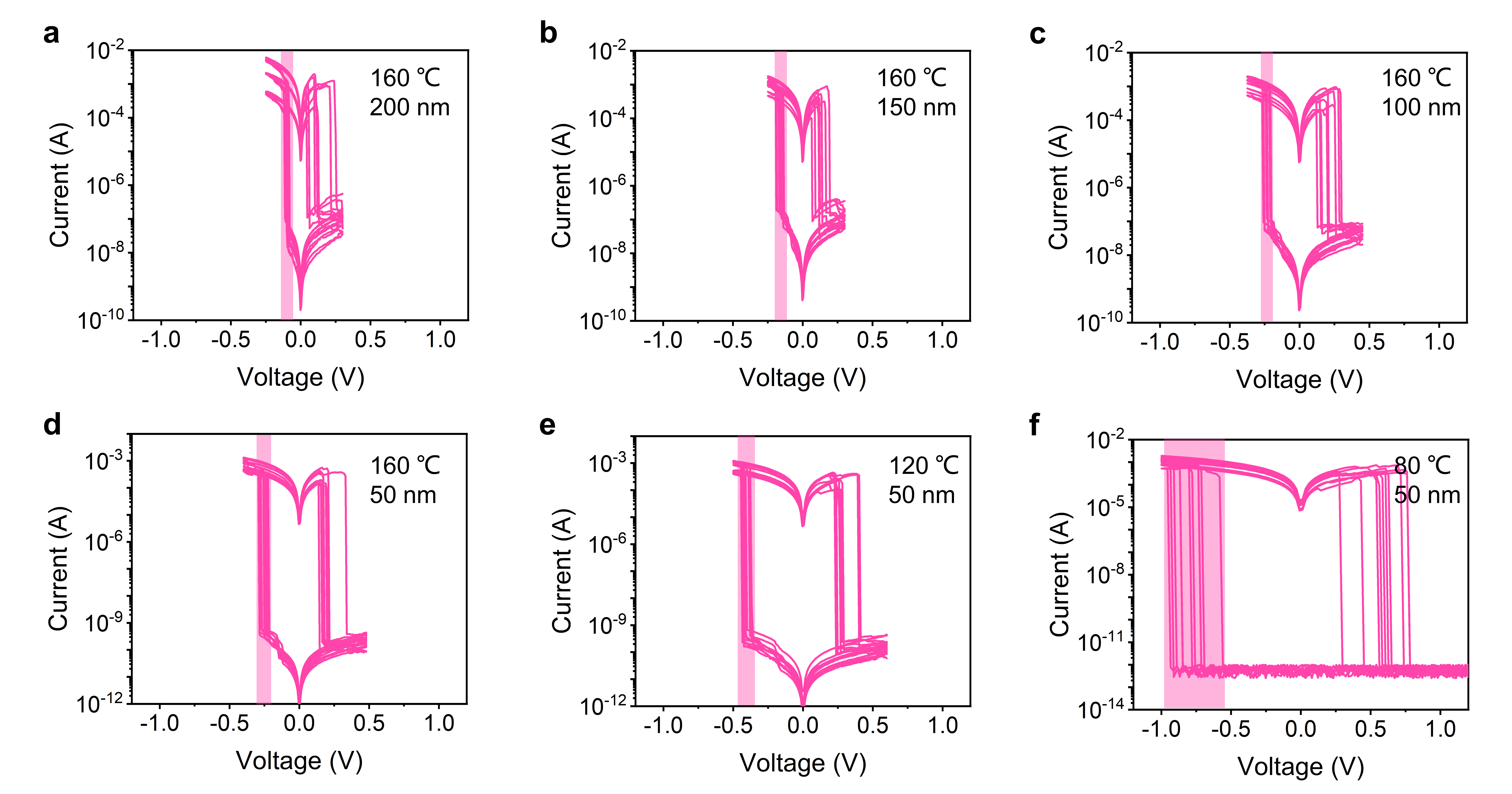


**Fig. S9** I-V characteristics of 10 devices for different batch. **a** 160 ℃, 200 nm Ag_2_S, **b** 160 ℃, 150 nm Ag_2_S, **c** 160 ℃, 100 nm Ag_2_S, **d** 160 ℃, 50 nm Ag_2_S, **e** 120 ℃, 50 nm Ag_2_S and **f** 80 ℃, 50 nm Ag_2_S


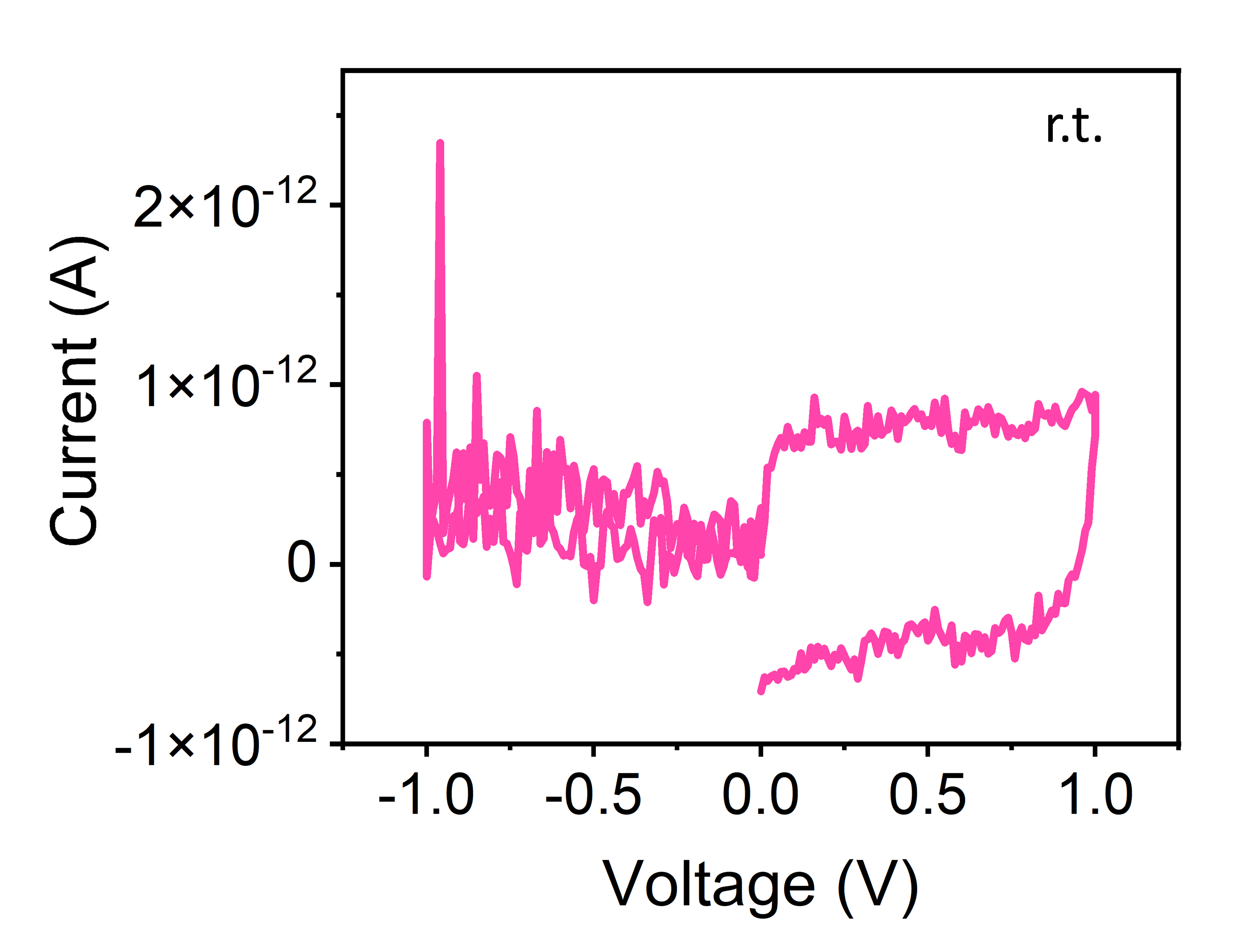


**Fig. S10** I-V characteristics of a Ag_2_S (50 nm)-based memristor annealed at room temperature. No resistance switching could be observed under ±1 V bias


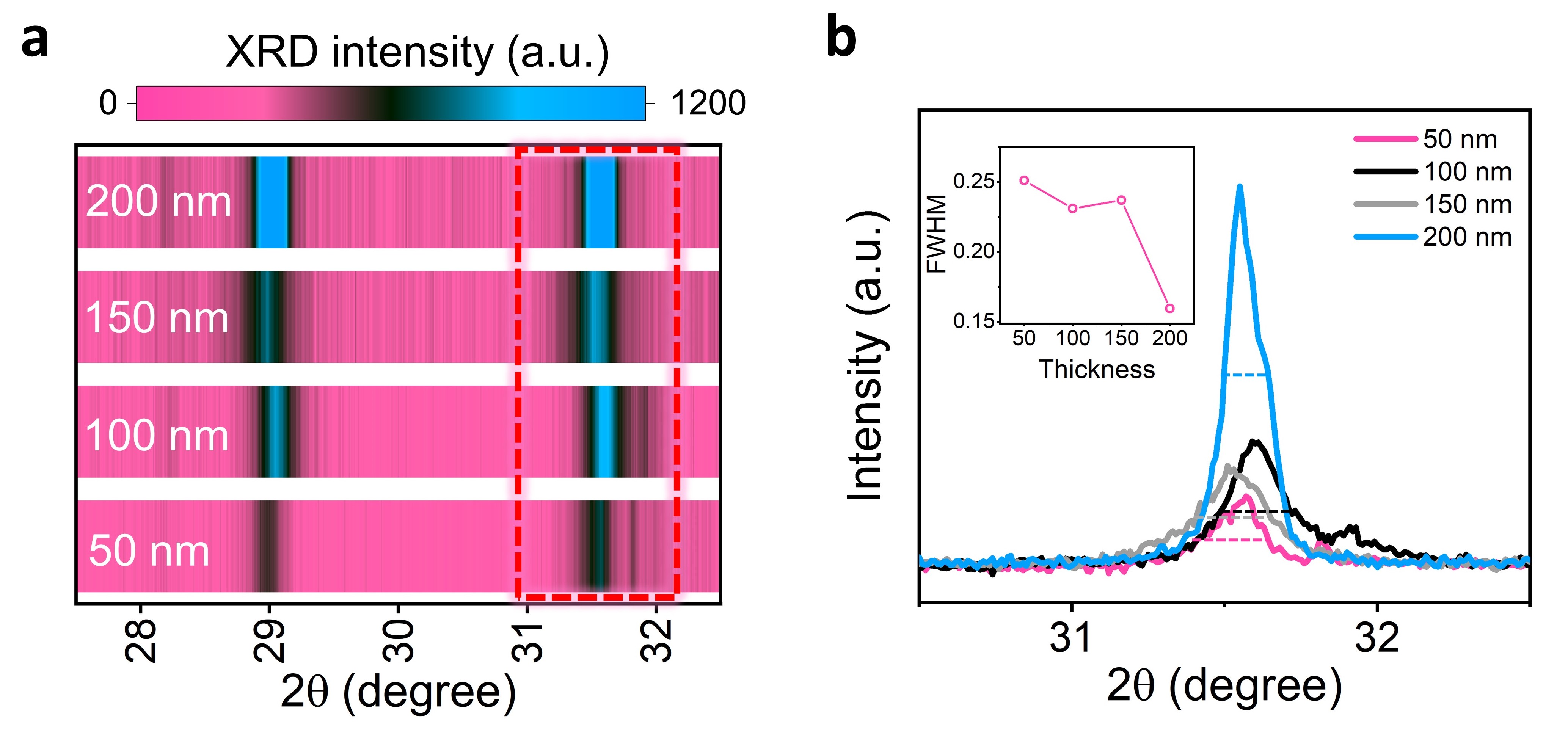


**Fig. S11** Threshold reduction by increasing the Ag_2_S film thickness. **a** X-ray diffractograms for Ag_2_S films (annealed at 160 ^o^C) with different thicknesses. **b** Full width at half maximum (FWHM) simulation using the diffraction singlet at 2θ ≈ 31.5^o^. The inset shows the calculated FWHM values as a function of the film thickness.


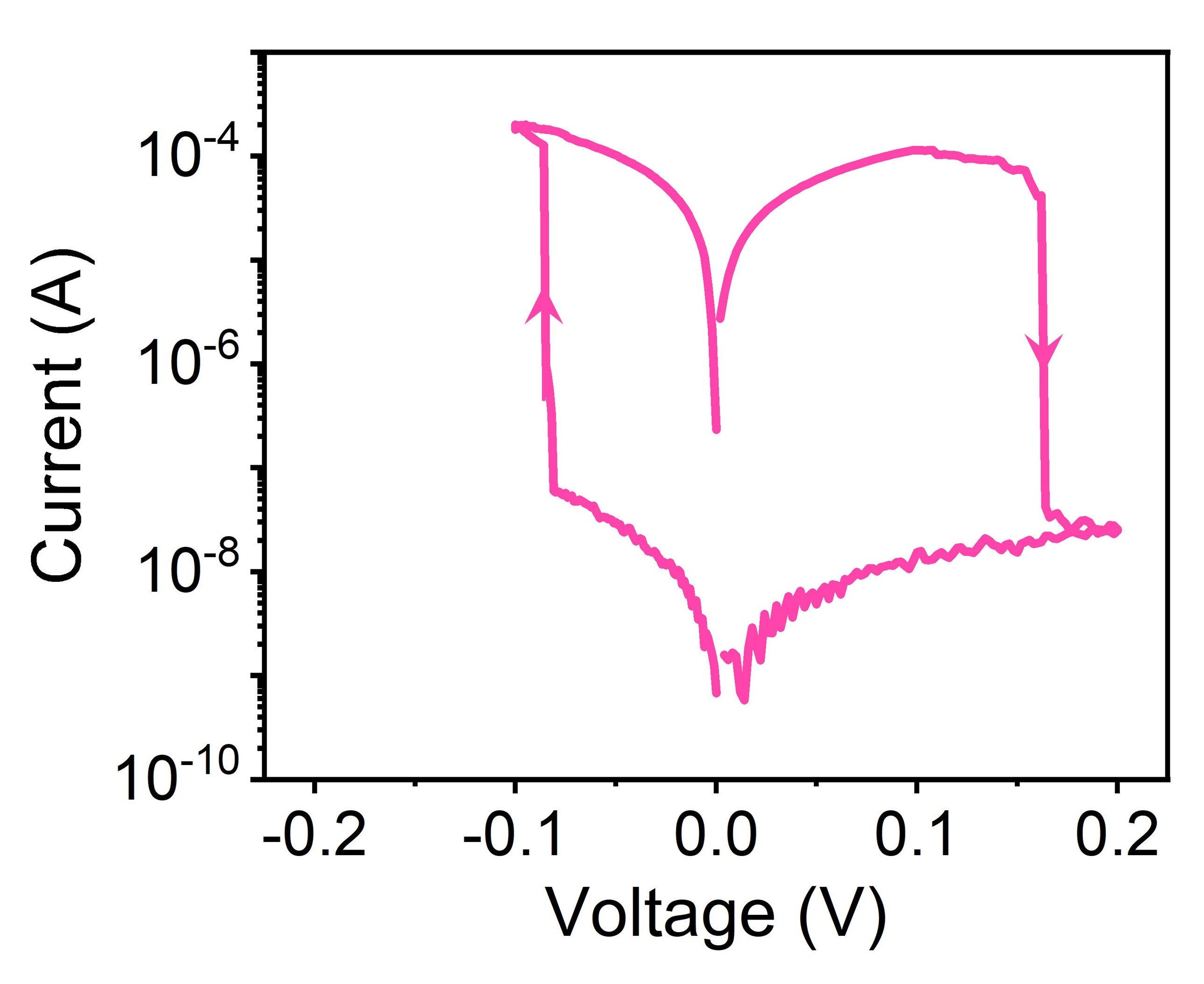


**Fig. S12** I-V characteristics of a device with a 250 nm-thick Ag_2_S film (160 ^o^C-annealed). The result shows a V_th_ of approximately -0.08 V.


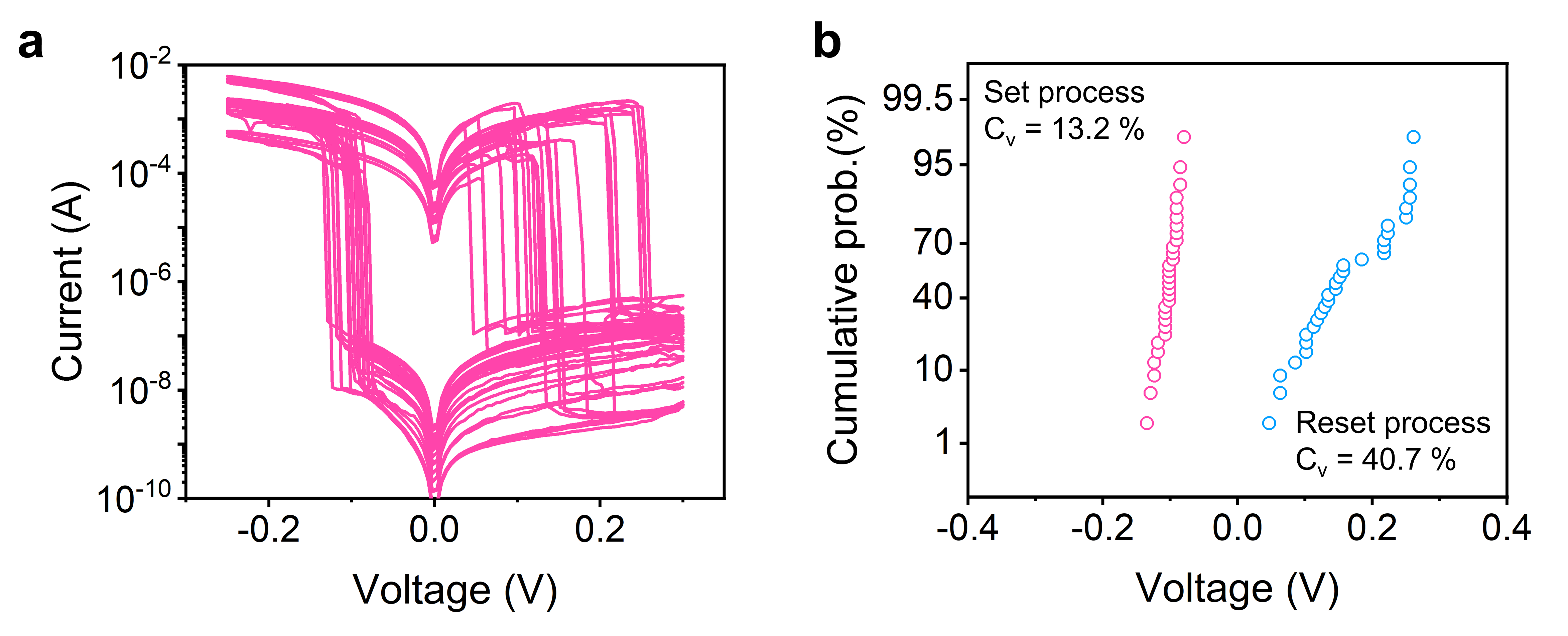


**Fig. S13** Switching behavior of different devices among the array. **a** I-V characteristics of 30 different Ag_2_S (200 nm, 160 ℃)-based devices. **b** Cumulative probability of V_th_ during set and reset processes from 30 devices


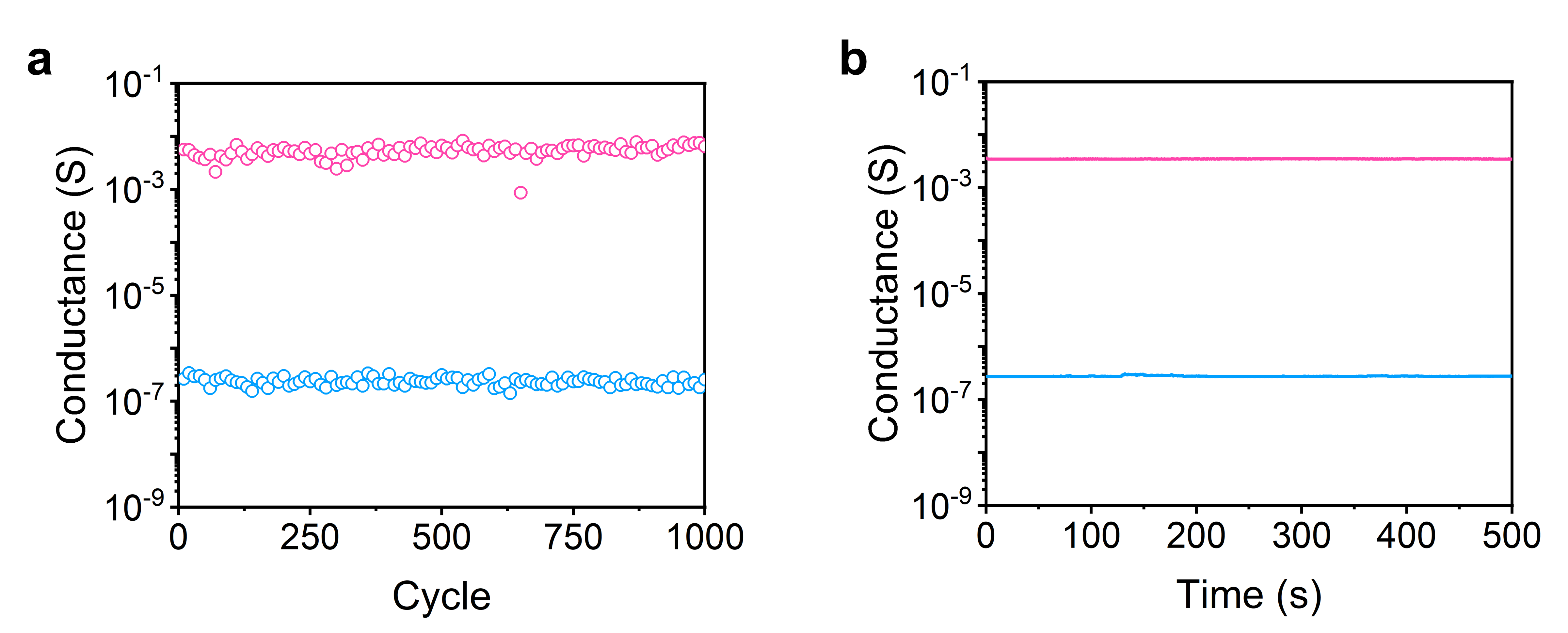


**Fig. S14** Switching behavior of the flexible Ag_2_S (200 nm, 160 ℃)-based device **a** The endurance of 10^3^ switching cycles under bending with 3 mm radius. The device was consecutively set/reset by -0.3 V, 10 ms/ 0.3 V,10 ms pulses, and the conductance was read at 5 mV. **b** The retention of the device (read at 5 mV) under bending with 3 mm radius.


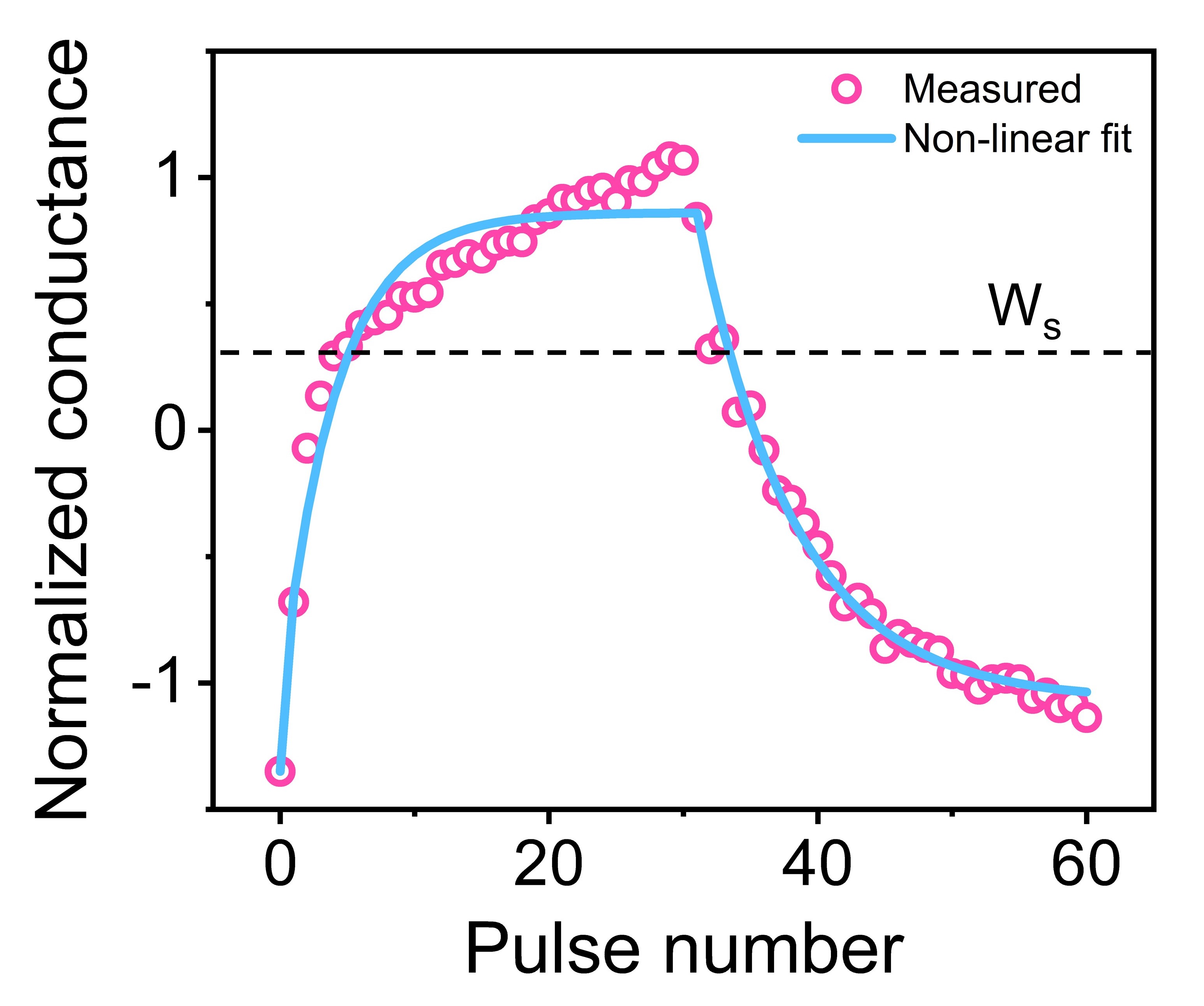


**Fig. S15** Non-ideal conductance modulation under identical pulses. The fitting of 30-cycle potentiation and depression processes using the previously reported method [4]. The obtained asymmetry of 0.25 was utilized for image classification simulation. The dashed line indicates the weight at the symmetric point (W_s_).


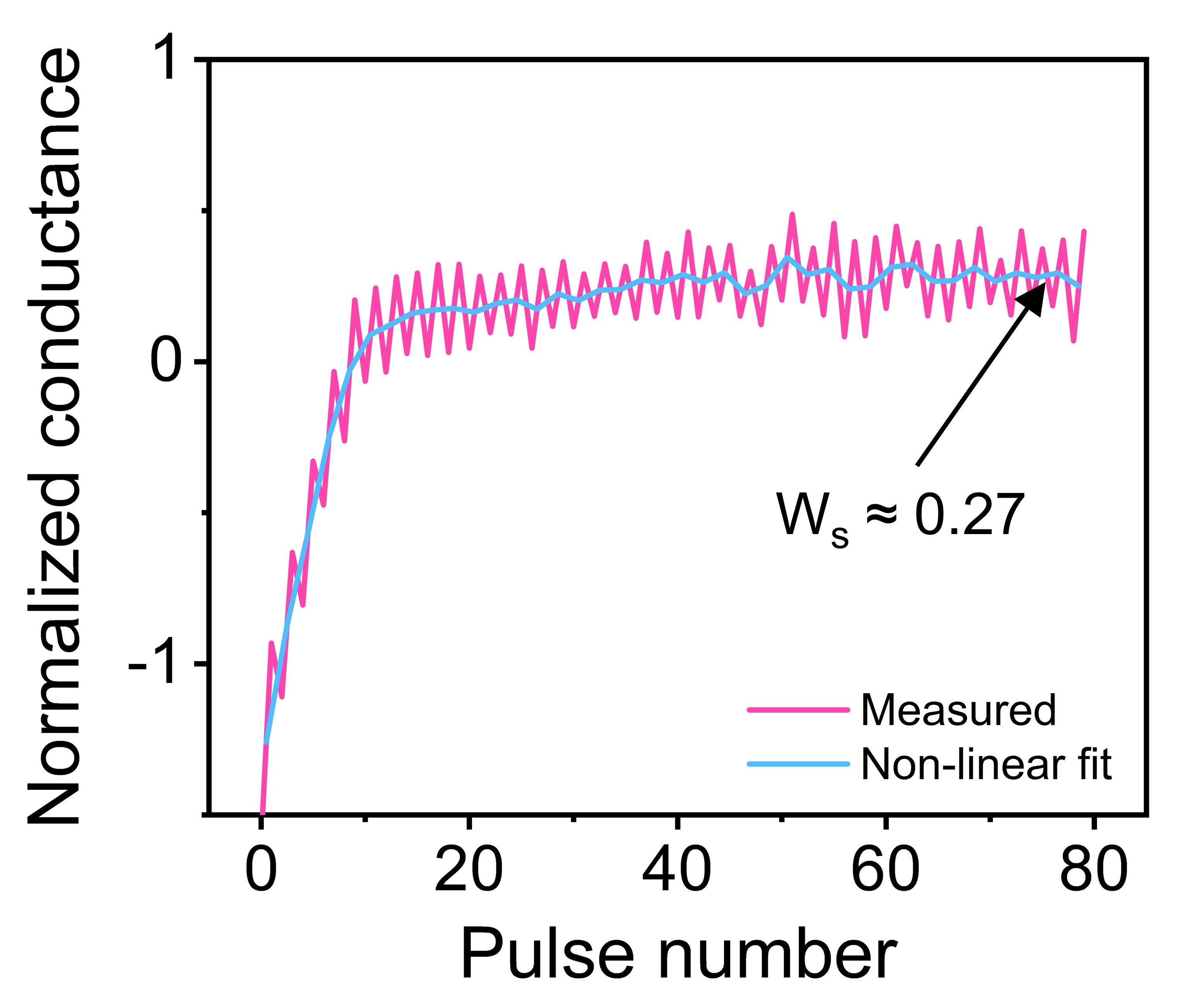


**Fig. S16** The symmetry point shifting technique. The normalized conductance of the Ag_2_S-based memristor under continuous up-down pulses (± 0.3 V, 1 μs). The results suggest a symmetric point with weight of ~0.27.

**Table S1.** Comparison of the switching energy of the Ag_2_S-based device and recently reported state-of-the-art memristors

| **Device structure** | **Device configuration** | **Active device area** | **Writing voltage** | **Pulse duration** | **Switching energy** | **Refs.** |
| --- | --- | --- | --- | --- | --- | --- |
| MoS_2_-based CBM | Single device | 0.1 mm^2^ | 0.066 V | / | / | [S5] |
| BiOI-based CBM | Single device | 250 µm^2^ | 0.5 V  (0.05V in d.c. tests) | 1 s | / | [S6] |
| CuZnS-based CBM | Textile-type | 1000 µm^2^ | 0.5 V (0.089 in d.c. tests) | 30 ns | 3.7× 10^-12^ J | [S7] |
| Ag/PMMA/CH_3_NH_3_PbI_3_/Au tip | Asymmetric tip array | 3um (radius) for Au tip | -0.11V in d.c. tests | / | / | [S8] |
| HfSe_2_-based RRAM | Crossbar array | 25 µm^2^ | 0.8 V | 50 ns | 8.2 × 10^-13^ J | [S9] |
| H_x_WO_3_-based ECRAM | Crossbar array | 30 µm^2^ | 4 V | 3 s | 1.0 × 10^-11^ J | [S1] |
| MoS_2_-based RRAM | Crossbar array | 0.63 µm^2^ | 60 V | 1 ms | 2.0 × 10^-12^ J | [S10] |
| Graphene/BN-based RRAM | Crossbar array | 1 µm^2^ | 10 V | 500 ns | 1.5 × 10^-9^ J | [S11] |
| WSe_2_-based RRAM | Crossbar array | 4900 µm^2^ | 0.5 V | 1 µs | 2.9 × 10^-12^ J | [S12] |
| HfO_x_-based RRAM | Crossbar array | 1. nm^2^ | >1.4 V | 5 ns | 1.0 × 10^-13^ J | [S13] |
| Ag_2_S-based CBM | Crossbar array | 25 µm^2^ | 0.3 V  (0.1 V in d.c. tests) | 1 µs | 1.5 × 10^-14^ J | This work |

**Supplementary References**

1. J. Cui, F. An, J. Qian, Y. Wu, L.L. Sloan et al., CMOS-compatible electrochemical synaptic transistor arrays for deep learning accelerators. Nat. Electron. **6**, 292–300 (2023). <https://doi.org/10.1038/s41928-023-00939-7>
2. T. Gokmen, W. Haensch Algorithm for training neural networks on resistive device arrays.: 1909.07908 (2019). <https://doi.org/10.3389/fnins.2020.00103>
3. T. Gokmen, Enabling training of neural networks on noisy hardware. Front. Artif. Intell. **4**, 699148 (2021). <https://doi.org/10.3389/frai.2021.699148>
4. N. Gong, M.J. Rasch, S.-C. Seo, A. Gasasira, P. Solomon et al., Deep learning acceleration in 14nm CMOS compatible ReRAM array: device, material and algorithm co-optimization. 2022 International Electron Devices Meeting (IEDM). December 3-7, 2022, San Francisco, CA, USA. IEEE, (2022)., 33.7.1–33.7.4.
5. P. Cheng, K. Sun, Y.H. Hu, Memristive behavior and ideal memristor of 1T phase MoS_2_ nanosheets. Nano Lett. **16**, 572–576 (2016). <https://doi.org/10.1021/acs.nanolett.5b04260>
6. P. Lei, H. Duan, L. Qin, X. Wei, R. Tao et al., High-performance memristor based on 2D layered BiOI nanosheet for low-power artificial optoelectronic synapses. Adv. Funct. Mater. **32**, 2201276 (2022). <https://doi.org/10.1002/adfm.202201276>
7. Y. Liu, X. Zhou, H. Yan, X. Shi, K. Chen et al., Highly reliable textile-type memristor by designing aligned nanochannels. Adv. Mater. **35**, e2301321 (2023). <https://doi.org/10.1002/adma.202301321>
8. J. Chen, Z. Feng, M. Luo, J. Wang, Z. Wang et al., High-performance perovskite memristor by integrating a tip-shape contact. J. Mater. Chem. C **9**, 15435–15444 (2021). <https://doi.org/10.1039/d1tc04164a>
9. S. Li, M.-E. Pam, Y. Li, L. Chen, Y.-C. Chien et al., Wafer-scale 2D hafnium diselenide based memristor crossbar array for energy-efficient neural network hardware. Adv. Mater. **34**, e2103376 (2022). <https://doi.org/10.1002/adma.202103376>
10. Lee, H.-S. et al. Dual-Gated MoS2 Memtransistor Crossbar Array. Adv. Funct. Mater. 30, 2003683 (2020). <https://doi.org/10.1002/adfm.202003683>
11. L. Sun, Y. Zhang, G. Han, G. Hwang, J. Jiang et al., Self-selective van der Waals heterostructures for large scale memory array. Nat. Commun. **10**, 3161 (2019). <https://doi.org/10.1038/s41467-019-11187-9>
12. Sivan, M. et al. All WSe_2_ 1T1R resistive RAM cell for future monolithic 3D embedded memory integration. Nat. Commun. 10, 5201 (2019). <https://doi.org/10.1038/s41467-019-13176-4>
13. B. Govoreanu, G.S. Kar, Y.-Y. Chen, V. Paraschiv, S. Kubicek et al., 10 × 10 nm^2^ Hf/HfO*_x_* crossbar resistive RAM with excellent performance, reliability and low-energy operation. 2011 International Electron Devices Meeting. December 5-7, 2011, Washington, DC, USA. IEEE, (2011)., 31.6.1–31.6.4.
